# Adaptive Genetic Divergence Despite Significant Isolation-by-Distance in Populations of Taiwan Cow-Tail Fir (*Keteleeria davidiana* var. *formosana*)

**DOI:** 10.3389/fpls.2018.00092

**Published:** 2018-02-01

**Authors:** Kai-Ming Shih, Chung-Te Chang, Jeng-Der Chung, Yu-Chung Chiang, Shih-Ying Hwang

**Affiliations:** ^1^Department of Life Science, National Taiwan Normal University, Taipei, Taiwan; ^2^Department of Geography, National Taiwan University, Taipei, Taiwan; ^3^Division of Silviculture, Taiwan Forestry Research Institute, Taipei, Taiwan; ^4^Department of Biological Sciences, National Sun Yat-Sen University, Kaohsiung, Taiwan

**Keywords:** adaptive divergence, fine-scale differentiation, *Keteleeria davidiana*, *K. davidiana* var. *formosana*, population genetics, SNP, species delimitation

## Abstract

Double digest restriction site-associated DNA sequencing (ddRADseq) is a tool for delivering genome-wide single nucleotide polymorphism (SNP) markers for non-model organisms useful in resolving fine-scale population structure and detecting signatures of selection. This study performs population genetic analysis, based on ddRADseq data, of a coniferous species, *Keteleeria davidiana* var. *formosana*, disjunctly distributed in northern and southern Taiwan, for investigation of population adaptive divergence in response to environmental heterogeneity. A total of 13,914 SNPs were detected and used to assess genetic diversity, *F*_ST_ outlier detection, population genetic structure, and individual assignments of five populations (62 individuals) of *K. davidiana* var. *formosana*. Principal component analysis (PCA), individual assignments, and the neighbor-joining tree were successful in differentiating individuals between northern and southern populations of *K. davidiana* var. *formosana*, but apparent gene flow between the southern DW30 population and northern populations was also revealed. Fifteen of 23 highly differentiated SNPs identified were found to be strongly associated with environmental variables, suggesting isolation-by-environment (IBE). However, multiple matrix regression with randomization analysis revealed strong IBE as well as significant isolation-by-distance. Environmental impacts on divergence were found between populations of the North and South regions and also between the two southern neighboring populations. BLASTN annotation of the sequences flanking outlier SNPs gave significant hits for three of 23 markers that might have biological relevance to mitochondrial homeostasis involved in the survival of locally adapted lineages. Species delimitation between *K. davidiana* var. *formosana* and its ancestor, *K. davidiana*, was also examined (72 individuals). This study has produced highly informative population genomic data for the understanding of population attributes, such as diversity, connectivity, and adaptive divergence associated with large- and small-scale environmental heterogeneity in *K. davidiana* var. *formosana*.

## Introduction

Conifers are reported to have slower evolutionary rate due to reduced levels of nucleotide mutation and large effective population size, but with higher ratio of non-synonymous to synonymous divergence, in comparison with angiosperms (Buschiazzo et al., 2012). Local adaptation in populations of coniferous species is not uncommon (e.g., Mimura and Aitken, 2010; Grivet et al., 2011; Chen et al., 2012; Fang et al., 2013). Limited dispersal shaping genetic structure of populations isolated geographically can result in a correlation between genetic and geographic distance known as isolation-by-distance (IBD) (Wright, 1943). However, adaptive divergence may occur between isolated populations because of topographical and ecological complexity known as isolation-by-environment (IBE), in which genetic distance is positively correlated with environmental distance (Wang et al., 2013; Sexton et al., 2014). The pattern of population divergence within a species can be either IBD, IBE, or both IBD and IBE, and IBD could be more prominent than IBE in plant species divergence (Sexton et al., 2014). Disentangling the effects of IBD from IBE is crucial to understanding their relative impact on population genetic structure, particularly because the relative contributions of IBD and IBE may vary among and within species (Wang et al., 2013; Sexton et al., 2014).

Most Taiwan endemic coniferous species are thought to be colonized from their ancestral species occurring in China with the exception of *Chamaecyparis formosensis* and *Ch. taiwanensis* (Wang et al., 2003; Chung et al., 2004; Chen et al., 2009; Chou et al., 2011). Genetic studies revealed congeneric sister species pair relationships of conifers occur in Taiwan and China, such as *Cunninghamia konishii* (Taiwan) and *Cu. lanceolata* (China) (Chung et al., 2004), *Calocedrus macrolepis* var. *formosana* (Taiwan) and *Ca. macrolepis* (China) (Chen et al., 2009), and *Taiwania cryptomeriodes* occurs in Taiwan colonized from China (Chou et al., 2011). *Ch. formosensis* and *Ch. taiwanensis* occur in Taiwan are known to be congeneric sister species pairs with *Ch. pisifera* and *Ch. obtusa* occur in Japan, respectively (Wang et al., 2003). Coniferous species endemic to Taiwan may display not only population adaptive divergence on island but also species level divergence with their ancestors. Genetic changes in response to local environments might have potential to keep pace with the rate of climate change (Robertson et al., 2014). Population structure of species may exhibit a pattern of IBE due to range expansion since colonization of their ancestral species and lead to locally adapted lineages associated with environmental heterogeneity (Holt, 2003; Schlotfeldt and Kleindorfer, 2006; Huang et al., 2015). Post-glacial expansion since the Last Glacial Maximum (LGM, 26.5–19.0 thousand years ago) (Lambeck and Chappell, 2001) may have also played a role in invoking locally adapted variants correlated with ecological complexity and climate change (Aitken et al., 2008; Chen et al., 2017).

Geographic isolation since the marine transgression following the LGM may induce higher probabilities of allopatric isolation between island-mainland sister species (Otte and Endler, 1989; Losos and Ricklefs, 2009). However, assumption of allopatric divergence between sister species of island and adjacent mainland since the isolation caused by the last marine transgression has been challenged (Li et al., 2010; Burridge et al., 2013). The level of allopatric divergence may be lower than expected because of prolonged gene flow between geographically isolated closely related species (Li et al., 2010) and also due to the recent colonization from mainland to island (Burridge et al., 2013). Species identification and characterization between closely related species have often been a challenge because of weak interspecific barriers. Low levels of genetic differentiation are frequently observed between Taiwan coniferous species and their adjacent mainland counterparts (Chung et al., 2004; Chou et al., 2011). In addition, multiple cycles of connection and isolation between Taiwan and adjacent large landmass would have led to high levels of interspecies gene flow causing difficulty in species delimitation (e.g., Chung et al., 2004; Worth et al., 2009; Strijk et al., 2012), and the differentiation of progenitor-derivative species pair may have restricted to some limited genomic hot-spots, while most of the genetic information are shared between sister species (Via, 2009; Wolf et al., 2010; Strasburg et al., 2012).

Populations of the warmth-loving Taiwan cow-tail fir (*Keteleeria davidiana* (Franchet) Beissner var. *formosana*) are presently disjunctly distributed on northern and southern rocky mountain ridges, respectively, at elevations of 300–600 m and 500–900 m (Li and Hsuan, 1994). The northern and southern populations of *K. davidiana* var. *formosana* occupy different environmental niches with varying floristic compositions (Chou et al., 2009). Taiwan cow-tail fir is thought to be derived from *K. davidiana* (Bertrand) Beissner that occurs in China (Farjon, 1989). The occurrence of *Keteleeria* since the Plio-Pleioscene boundary was revealed by a pollen record and in minor proportion during the early Pleistocene Praetiglian, late Pleistocene, and early Holocene around 10,000 years ago in central Taiwan (Tsukada, 1967). The disappearance of *Keteleeria* from areas other than northern and southern Taiwan may have resulted from its recalcitrant seed storage behavior (Yang et al., 2006) and low natural regeneration rate (Wang, 1987) that rendered *Keteleeria* incompetent in competition with rapidly growing subtropical species such as *Machilus* and *Castanopsis* (Su, 1984). In addition, human disturbance may also have contributed to the disappearance of *K. davidiana* var. *formosana* from parts of its former range.

Restriction site-associated DNA sequencing (RADseq) (Baird et al., 2008) and its related methodologies, such as genotyping-by-sequencing (Elshire et al., 2011) and double digest RADseq (ddRADseq) (Peterson et al., 2012) are powerful methods in genotyping thousands of genomic markers distributed randomly across genome. These techniques, share the common features of using one or more restriction enzymes to sample a subset of genomic loci, are applicable in population genetics study on species with no reference sequence information (Davey and Blaxter, 2010). RADseq and related techniques can sample genomic variation at reduced complexity from many individuals particularly for non-model organisms at reasonable cost, and are important technologies for ecological, evolutionary, and conservation genomics (Hoffmann et al., 2015; Andrews et al., 2016). RADseq related techniques can be useful in examining natural population adaptive divergence (Parchman et al., 2012; Pannell and Fields, 2014) and in revealing candidate genome regions that involved in speciation (Eaton and Ree, 2013; Sobel and Streisfeld, 2015).

In a previous study based on amplified fragment length polymorphism (AFLP), local adaptation was found in *K. davidiana* var. *formosana* (Fang et al., 2013). However, no clear genetic distinction between disjunctly distributed northern and southern populations was found except when the *F*_ST_ outliers potentially evolved under selection were used in the analysis. One of the main findings of the previous study was the associations of environmental variables, such as temperature, precipitation, and humidity, with *F*_ST_ outliers, indicating IBE between disjunctly distributed northern and southern populations of *K. davidiana* var. *formosana*. However, because of geographically distant distributions of the northern and southern populations, IBD can also be important influencing population genetic structure. In the present study, we employed ddRADseq in genotyping samples of *K. davidiana* var. *formosana* to obtain genome-wide single nucleotide polymorphism (SNP) markers for the purpose of investigating population adaptive divergence in *K. davidiana* var. *formosana*. We hypothesized the occurrence of IBE as well as IBD because of habitat heterogeneity and geographic isolation, in particular, between northern and southern populations of Taiwan cow-tail fir. The relative importance of geography and environment shaping the patterns of genetic variation was assessed to gain a deeper understanding of how environmental factors influence evolutionary processes. We also aimed to test the selection of SNPs closely associated with environmental variables across populations of *K. davidiana* var. *formosana* and to examine whether potential selective outliers link to specific gene functions that may have played significant roles underlying local adaptation. To examine the genetic relationships between populations of *K. davidiana* var. *formosana* and *K. davidiana*, samples of *K. davidiana* were also genotyped based on ddRADseq.

## Materials and methods

### Sample collections and DNA extraction

**Figure 1:**
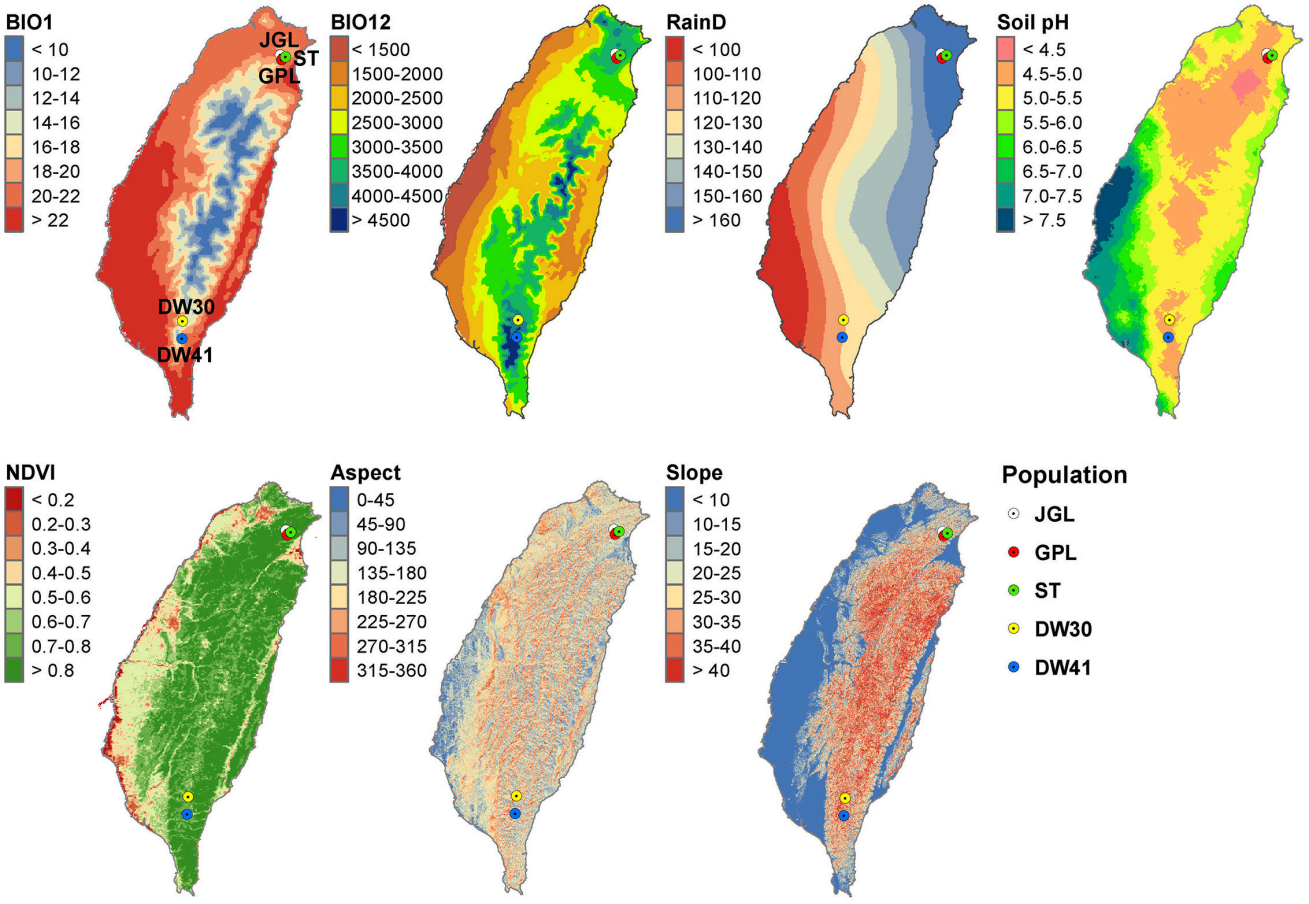
Geographic distribution of the five populations of Taiwan cow-tail fir and annual mean gradients of seven environmental variables. *BIO1*, annual mean temperature; *BIO12*, annual precipitation; *RainD*, number of rainfall days per year. Annual mean gradients were smoothed using a universal spherical model of the Kriging method in ArcGIS.

Samples of endangered *K. davidiana* var. *formosana* were collected, including three northern and two southern populations (Table 1, Figure 1). The number of old growth trees, ages between 100 and 300 years, were ranged between 20 and 81 for Taiwan cow-tail fir in natural stands (Chung, J.-D., unpublished data). The distances between pairs of the three northern populations are within 1–3 km, and they are distantly separated from the two southern populations (263–290 km). Distance between the two southern populations is about 26 km. Approval of sample collection was granted by the Forestry Bureau, Council of Agriculture, Taiwan (permit number: 101-AgroScience-1.1.2-B-e1). Genomic DNA was extracted from *K. davidiana* (*n* = 10, Kunming Botanical Garden, Yunnan, China) and *K. davidiana* var. *formosana* (*n* = 62) using a modified cetyl trimethyl ammonium bromide (CTAB) protocol (Dehestani and Kazemitabar, 2007) and quantified with a Nanodrop 1000 Spectrophotometer (Thermo Fisher Scientific, Waltham, MA, USA).

**Table 1:** Population genetic parameters of the five sampled populations of Taiwan cow-tail fir based on ddRADseq.

**Population**	**Elevation (m)**	**Number of individuals**	**Latitude/longitude**	***A*_R_ (SE)**	**π (SE)**	***H*_O_ (SE)**	***H*_E_ (SE)**	***uH*_E_ (SE)**	***F*_IS_ (95% CI)**	***I*_A_ (*P*)**	***r*_D_ (*P*)**
JGL	376	10	24°54′52.278″N	1.105 (0.001)	0.109 (0.001)	0.135 (0.002)	0.100 (0.001)	0.109 (0.001)	−0.270^*^ (−0.282, −0.258)	20.804 (1.000)	0.004 (1.000)
			121°40′36.679″E								
GPL	561	8	24°53′52.959″N	1.104 (0.001)	0.109 (0.001)	0.135 (0.002)	0.099 (0.001)	0.109 (0.001)	−0.264^*^ (−0.278, −0.251)	4.252 (0.148)	0.001 (0.106)
			121°41′13.058″E								
ST	436	10	24°53′35.246″N	1.105 (0.001)	0.109 (0.001)	0.133 (0.002)	0.101 (0.001)	0.109 (0.001)	−0.2434^*^ (−0.257, −0.230)	47.340 (1.000)	0.008 (1.000)
			121°41′48.236″E								
DW30	799	17	22°36′42.394″N	1.094 (0.001)	0.096 (0.001)	0.119 (0.002)	0.092 (0.001)	0.096 (0.001)	−0.249^*^ (−0.264, −0.233)	23.811 (0.459)	0.004 (0.380)
			121°0′19.435″E								
DW41	702	17	22°25'38.369″N	1.091 (0.001)	0.093 (0.001)	0.119 (0.002)	0.089 (0.001)	0.093 (0.001)	−0.298^*^ (−0.312, −0.284)	4.931 (0.002)	0.001 (0.002)
			120°51′3.006″E								
Average				1.100	0.103	0.128	0.096	0.103			

*N, Number of samples; A_R_, mean allelic richness; π, nucleotide diversity; H_O_, observed heterozygosity; H_E_, expected heterozygosity; I_A_, index of association; r_D_, modified index of association*.

* *P < 0.0001*.

### ddRADseq library construction and sequencing

ddRADseq libraries were prepared following Peterson et al. (2012) with modifications. Genomic DNA was double digested with *Eco*RI (20 units) and *Mse*I (20 units) and ligated to barcoded P1 and indexed P2 adapters binding to *Eco*RI and *Mse*I overhangs, respectively. Fragments in the 250–450 bp size range were selected from an agarose gel. Amplified fragment libraries were quantified using quantitative real time polymerase chain reaction (qPCR) and pooled in equimolar amounts for sequencing on the Illumina HiSeq2000 platform (Illumina, San Diego, CA, USA) with 36 samples per lane. Sequence reads, 85 bp in read 1 and 90 bp in read 2, were assigned to individual samples based on barcode sequences. The ddRADseq library construction, sequencing, and the following processing of sequence reads were conducted at the Beijing Genomic Institute, China.

### ddRAD sequence analysis

Restriction site sequences on both paired-end (PE) reads were removed using FASTX-Toolkit v.0.0.13 (http://hannonlab.cshl.edu/fastx_toolkit/). The two base pairs following *Eco*RI restriction sites were removed avoiding the effect of GC content asymmetry that may cause problem in the subsequent *de novo* assembly (Kozarewa et al., 2009). A 9-bp sequence following *Mse*I restriction sites was also removed to generate equal length in the two PE reads. Moreover, a 5-bp C and a 5-bp T, respectively, was added to the end of read 1 and read 2, resulting in 78 bp in both reads, using a php script (Supplementary File 1) considering that the two PE reads with high sequence similarity will not be treated as same locus during *de novo* assembly.

STACKS software pipeline v.1.40 was used for read filtering and SNP genotyping (Catchen et al., 2011). Using FASTQC v.0.11.2 (Andrews, 2010), low quality sequence reads with a Phred33 quality score < 10 (as suggested by the authors of STACKS) were discarded and any reads with an uncalled base was also removed using the “process_radtags” module. The “ustacks,” “cstacks,” “sstacks,” and “populations” modules of STACKS were used to obtain final genotypes called at a Minor Allele Frequency of 5%. The number of SNP obtained was used to determine the values of parameters m, M, and N for “ustacks” module and of parameter n for “cstacks” module (Paris et al., 2017). The settings of these parameters are known to influence the number of SNP obtained, estimation of population genetic diversity measures, and downstream population genetic inference (Mastretta-Yanes et al., 2015; Paris et al., 2017; Shafer et al., 2017). Three individuals from each of the five populations of *K. davidiana* var. *formosana* and *K. davidiana* were used to compute number of SNP (in two of three samples in each population) obtained and percentage of polymorphic loci (Supplementary Table 1; Paris et al., 2017). Parameter m was evaluated from 3 to 5, and m = 3 had the highest number of SNP obtained. Parameter M was evaluated from 0 to 5. Parameter N was set to M+2 (Paris et al., 2017). The number of SNP obtained increased by increasing M and reached a plateau between M = 2 and M = 4, but dropped apparently when M = 5. When M and n were set to 2 and 1, respectively, no apparent change in percentage of polymorphic loci was found among different values of m. No apparent change in percentage of polymorphic loci was also found among different values of M when m and n were set to 3 and 1, respectively. Therefore, the values of parameters m, M, N, and n were set to 3, 2, 4, and 1, respectively, for STACKS pipeline. The “cstacks” module created a catalog of SNPs, which was used to genotype each individual with the “sstacks” module. In “populations” module, we obtained three data sets that allowed the minimum proportions of non-missing genotypes at 40, 50, and 60% of samples across populations with multiple SNPs per stack, respectively.

### Environmental variables

Environmental variables include 19 bioclimate, 10 ecological, and three topological variables. Bioclimate data at 30 s spatial resolution (~1 km) were downloaded from the WorldClim v.1.4 (http://www.worldclim.org/; Hijmans et al., 2005). Monthly mean values of ecological variables at spatial resolution of 1 km included relative humidity, cloud cover, time of sunshine, wet days (number of days with >0.1 mm of rain per month), number of rainfall days per year, and mean wind speed were obtained from the Data Bank for Atmospheric Research (DBAR, http://www.narlabs.org.tw/en/, recorded in 1990–2013) using a universal spherical model of the Kriging method in ArcGIS (Chang et al., 2014). Remote sensing data based on moderate resolution imaging spectroradiometer (MODIS) for ecological factors included normalized difference vegetation index (NDVI) and enhanced vegetation index were obtained from Land Process Distributed Active Archive Center (http://lpdaac.usgs.gov). Monthly MODIS images were generated based on a maximum values composite procedures (Huete et al., 2002). Soil pH values of sample sites were acquired from the Agriculture and Food Agency of Taiwan based on an island-wide soil investigation (*n* = 1,150) conducted in 1969–1986 (Chang et al., 2009). Annual moisture index (Thornthwaite, 1948) was also calculated for each sample site. Topographic variables, including aspect, elevation, and slope, were derived from a 40 m resolution digital terrain model, and monthly mean values for sampling sites were computed in ArcGIS (Chang et al., 2014). Variance inflation factor (VIF) was computed for environmental variables using the “vif” function of usdm package (Naimi et al., 2014) in the R environment (R Development Core Team, 2013). Correlation coefficient and VIF were calculated for the three sets of environmental variables (bioclimate, ecological, and topological variables) separately, and environmental variables with VIF > 20 (Borcard et al., 2011) and highly correlated with other variables (|r| > 0.8) were removed. Seven environmental variables were retained, including bioclimate: annual mean temperature and annual precipitation; ecology: number of rainfall days per year, Soil pH, and NDVI; and topology: aspect and slope (Supplementary Table 2).

### Detection of *F*_ST_ outliers and association with environmental variables

BAYSECAN v.2.1 (Foll and Gaggiotti, 2008) was used to identify *F*_ST_ outliers. BAYESCAN estimates the posterior odds (PO), the ratio of posterior probabilities of selection over neutrality. The parameters for running BAYESCAN were a 100 pilot runs of 50,000 iterations and followed by a sample size of 50,000 with thinning interval of 20 among 10^6^ iterations. Any SNP with log_10_(PO) > 0.5 was considered to have substantial evidence for selection (Jeffreys, 1961). FDIST within the LOSITAN workbench (Beaumont and Nichols, 1996; Antao et al., 2008) was also used to identify outliers potentially evolved under selection. In FDIST, outliers were identified by comparing observed distribution of *F*_ST_ conditioned on expected heterozygosity with neutral expectations at a 99.5% confidence interval (CI) and a false discovery rate (FDR) of 1% with each run comprising 10^6^ simulations with both “neutral mean *F*_ST_” and “force mean *F*_ST_” selected, and removed *F*_ST_ outliers to increase the reliability when calculating the global distribution of *F*_ST_. Loci that are detected as outliers by both BAYESCAN and FDIST were analyzed with Samβada (Stucki et al., 2014) for the associations between all possible pairs of allele frequencies of SNPs and environmental variables using multiple univariate logistic regression. The 23 outlier SNPs identified by BAYESCAN and FDIST (see Results) were coded for allelic presence (“1”) or absence (“0”), producing 69 SNP genotypes (i.e., “00,” “01,” and “11” for each of the 23 outlier SNPs). These genotypes were tested for associations with the seven environmental variables retained (Supplementary Table 2), resulting in 483 tests. Models including and excluding the environmental variables were compared, and significant fit was identified based on both Wald and G scores with an FDR cutoff of 0.01. Three data sets of genetic variation were generated, including total, neutral, and outlier data sets, after identification of *F*_ST_ outliers. Sequences containing outlier SNPs were searched (BLASTN) against the NCBI non-redundant nucleotide database for potential gene region identification. In BLASTN, an *E*-value of 0.001 was used as threshold for significant sequence similarity. Pairwise linkage disequilibrium (LD) between outlier SNPs was assessed using a two-locus exact test implemented in ARLEQUIN v.3.5, and significance determined by 10,000 permutations (Excoffier and Lischer, 2010).

### Genetic diversity, structure, and relationships

Data with missing values may influence the individual assignment and phylogenetic analysis using reduced representation of RADseq data (Chattopadhyay et al., 2014; Huang and Knowles, 2016). The number of SNPs retained by STACKS based on the extent of missing data vary dramatically (Supplementary Table 3). In the present study, different percentages of missing value (SNPs found in at least 40, 50, and 60% of samples across populations) data sets were generated. These data sets were evaluated for the potential effects on distributions of the levels of population genetic diversity measures and pairwise locus *F*_ST_ using Kolmogorov Smirnov (KS) test (the “ks.test” function of R). Data of 50% missing value across populations was adopted, based on the number of SNP obtained (Supplementary Table 3), and used for all the following analyses. We calculated nucleotide diversity (π), observed (*H*_O_), and expected (*H*_E_) heterozygosity using STACKS. Nei's unbiased *H*_E_ (*uH*_E_) was also calculated (Nei, 1978). Pairwise locus *F*_ST_ was calculated with the “popgenreport” function of R package PopGenReport (Adamack and Gruber, 2014). CI (95%) of *F*_IS_ for each population was calculated using “boot.ppfis” function of R package hierfstat with 999 bootstrap resampling (Goudet, 2005), and *P*-values calculated. Mean allelic richness (i.e., mean number of alleles per locus, *A*_R_) was calculated with the function “allel.rich” of R package PopGenReport. Proportion of shared alleles between species and between populations was calculated with the “pairwise.propShared” function of R package PopGenReport. Index of association (*I*_A_) (Brown et al., 1980) and modified index of association (*r*_D_) (Agapow and Burt, 2001) represent multilocus LD were calculated using the “ia” function of R poppr package (Kamvar et al., 2014), and significance of non-zero *I*_A_ and *r*_D_ values was tested with 999 permutations.

We used linear mixed-effects models with maximum likelihood (ML) estimation using the “lmer” function of R package lme4 (Bates et al., 2015) to assess whether genetic diversity measures (*A*_R_, π, *H*_O_, *H*_E_, and *uH*_E_) were significantly different between populations of *K. davidiana* var. *formosana*. Population and locus were used as fixed and random effects, respectively, in linear mixed-effects models. Overall difference was tested using the “Anova” function of R package car (Fox and Weisberg, 2011) based on the type-II Wald χ^2^-test, and Tukey's *post-hoc* pairwise comparisons were performed using the “lsmeans” function of R package lsmeans (Lenth, 2016).

Three data sets, including total, neutral, and outlier, were used for computation of genetic differentiation via analysis of molecular variance (AMOVA) and across population *F*_ST_. We performed AMOVA using the “poppr.amova” function of R package poppr and significance tested with the “randtest” function of R package ade4 (Dray and Dufour, 2007). Across population *F*_ST_ was calculated using the “popStructTest” function of R package strataG (Archer et al., 2017) and tested the significance (999 permutations). For genetic assignment of individuals, only the total data was used. Estimation of individual ancestries was performed with ADMIXTURE v.1.3 based on ML method (Alexander et al., 2009). We ran ADMIXTURE for each *K* from *K* = 1 to *K* = 5 (*K. davidiana* var. *formosana* only) and from *K* = 1 to *K* = 6 (both *K. davidiana* and *K. davidiana* var. *formosana*) using the default settings, and the best *K* evaluated with 10-fold cross-validation (CV) procedure. Genetic assignment of individuals was also inferred based on sparse non-negative factorization (sNMF) and least-squares optimization with the “snmf” function of R package LEA (Frichot and Francois, 2015). The snmf settings were: regularization parameter = 100, iterations = 200, and repetitions = 10 with other arguments set to defaults, and the best *K* evaluated with the means of minimal cross-entropy (CE).

For principal component analysis (PCA), allelic frequency data was first generated with the “makefreq” function of R package adegenet (Jombart and Ahmed, 2011) with missing values replaced with the mean of the corresponding allele and analyzed using the “prcomp” function of R based on correlation matrix. A neighbor joining (NJ) tree was generated based on Nei's genetic distance (Nei, 1978), and bootstrap support value (BSV) was calculated using the “aboot” function of R package poppr with 1,000 bootstrap resampling, missing values were also replaced with the mean of the corresponding allele in the NJ tree construction.

### Importance of environmental variables explaining genetic variation

The most important environmental variables explaining genetic variation based on the total and outlier data sets were analyzed according to the double stopping criterion (Blanchet et al., 2008) using the “forward.sel” function of R package packfor (Dray, 2013). Significance was determined using 999 permutations. In the forward selection analysis, three categories (bioclimate, ecology, and topology) of explanatory variables were used separately.

### Isolation-by-environment and isolation-by-distance

Mantel correlation between geographic distance and environmental heterogeneity was assessed with the “mantel” function of R package vegan (Oksanen et al., 2017). Environmental and geographic distance matrices were generated using the “dist” function of R based on Euclidean distance. Nei's genetic distance matrix (Nei, 1978) was calculated using the “nei.dist” function of R package poppr. The relative role of environment and geography on population genetic distance was evaluated using multiple matrix regression with randomization (MMRR) implemented in the “MMRR” function of R (Wang, 2013) based on the total and outlier data sets. MMRR was used to quantify how dependent variable (genetic variation) responds to changes in explanatory variables (environmental, geographic, and environmental plus geographic). In MMRR, regression coefficients IBE (β_E_) and IBD (β_D_) were calculated and tested with 999 permutations.

## Results

### Genotyping and SNP calling

A total of 582,985,540 PE reads were obtained following ddRADseq of 72 individuals of *K. davidiana* and *K. davidiana* var. *formosana* (Supplementary Table 4). After filtering using STACKS pipeline to remove low quality reads, ambiguous barcodes, and overrepresented sequences, 574,480,673 reads remained. A catalog containing 7,663,569 loci was created for generation of genotypes in all individuals. For each individual, an average number of 395,546 stacks were assembled with an average read depth of 5.29 per stack. Further filtering with the presence of SNPs in at least 50% of examined samples across populations resulted in a total of 17,982 SNPs genotyped for samples included both *K. davidiana* and *K. davidiana* var. *formosana* (Supplementary Table 3). When only samples of *K. davidiana* var. *formosana* were used, we obtained 13,914 SNPs.

We also obtained the 40 and 60% missing data sets and examined the effect of the levels of missing values on the distributions of the levels of population genetic diversity measures and pairwise locus *F*_ST_. The distributions of the levels of genetic diversity measures across populations and distributions of pairwise locus *F*_ST_ for the three missing data sets are shown in Supplementary Figures 1, 2. KS test revealed that the distributions of population genetic diversity measures across populations and pairwise locus *F*_ST_ differed significantly between 40 and 60% missing data sets (Supplementary Figures 1, 2; Supplementary Table 5). Significant shape shifting between the distributions of pairwise locus *F*_ST_ of 40 and 50% missing data sets was revealed (*P* = 0.038). KS test showed no significant differences between 40 and 50% and between 50 and 60% missing data sets in the distributions of π, *H*_O_, *H*_E_, and *uH*_E_ across populations. However, the distributions of *A*_R_ across populations were significantly different between 50 and 60% missing data sets probably due to the significant increase in the number of different alleles per locus in 60% than in 50% missing data set (Supplementary Figure 1). In addition, 60% missing data set had much lower numbers of SNPs compared with 50% missing data set (Supplementary Table 3) and may cause the loss of valuable information in individual assignments (Huang et al., 2010; Chattopadhyay et al., 2014) and removing SNPs that with high mutation rate for recent divergence (Huang and Knowles, 2016). In contrast, 40% missing data set that with higher number of missing values may also influence individual assignments (Chattopadhyay et al., 2014; Huang and Knowles, 2016). We chose to adopt the 50% missing data set, based on number of SNP obtained (Supplementary Table 3), for further use in the present study. All raw sequences are available at NCBI SRA Bioproject PRJNA419582 and Biosample SAMN08093166–SAMN08093171. The 50% missing data sets included both investigated species and *K. davidiana* var. *formosana* only in STRUCURE format (Pritchard et al., 2000) are provided in Data Sheets 1, 2, respectively.

### Population genetic diversity of taiwan cow-tail fir

We found average values of all population genetic diversity measures were smaller in *K. davidiana* var. *formosana* (*A*_R_ = 1.078, π = 0.080, *H*_O_ = 0.100, *H*_E_ = 0.075, and *uH*_E_ = 0.080) than the values in *K. davidiana* (*A*_R_ = 1.096, π = 0.101, *H*_O_ = 0.121, *H*_E_ = 0.093, and *uH*_E_ = 0.100) (Supplementary Table 6). Population measures of genetic diversity within *K. davidiana* var. *formosana*, including *A*_R_, π, *H*_O_, *H*_E_, and *uH*_E_, averaged 1.100 (1.091–1.105), 0.103 (0.093–0.109), 0.128 (0.119–0.135), 0.096 (0.089–0.101), and 0.103 (0.093–0.109), respectively (Table 1). Overall, genetic diversity measures within *K. davidiana* var. *formosana* differed significantly among populations (type-II Wald χ^2^-test, *A*_R_: χ^2^ = 334.25; π: χ^2^ = 436.85; *H*_O_: χ^2^ = 359.03; *H*_E_: χ^2^ = 254.86; and *uH*_E_: χ^2^ = 436.9, and all *P* < 0.0001). *H*_E_ was lower compared to *H*_O_ in all populations (25.4, 26.1, 24.1, 22.6, and 25.3% lower, respectively, for populations JGL, GPL, ST, DW30, and DW41). The levels of genetic diversity measures (*A*_R_, π, *H*_O_, *H*_E_, and *uH*_E_) were comparatively higher in the northern than in the southern populations (Table 1, Tukey's *Ps* < 0.0001). No difference was found between the three northern populations in all genetic diversity measures. The levels of all genetic diversity measures except *H*_O_ were also significantly different between the two southern populations (*A*_R_: *P* = 0.026; π: *P* = 0.027; *H*_O_: *P* = 0.999; *H*_E_: *P* = 0.033; and *uH*_E_: *P* = 0.027). *F*_IS_-values were all negative and deviate significantly from zero for all populations either with data included only *K. davidiana* var. *formosana* (Table 1) or data included both *K. davidiana* var. *formosana* and *K. davidiana* (Supplementary Table 6). Multilocus LD assessed using *I*_A_ and *r*_D_ found only the DW41 population had significant non-zero values (*I*_A_ = 4.931, *P* = 0.002; *r*_D_ = 0.001, *P* = 0.002, Table 1) than expected under a null distribution, indicating significant non-random associations between alleles in the DW41 population.

### Potential selective outliers in taiwan cow-tail fir

Twenty-three outlier SNPs (0.2%) were found using both BAYESCAN and FDIST in global and pairwise population comparisons (Table 2), and 15 of them were found to be associated strongly with environmental variables, including annual mean temperature, number of rainfall days per year, aspect, and soil pH using Samβada (Table 2). Most of these 15 outlier SNPs had log_10_(PO) > 1.0 in either global or between population comparisons indicating strong evidence for selection (Jeffreys, 1961), and outlier SNPs that had log_10_(PO) = 1,000 were observed when the southern DW41 population was compared to the northern populations. Samβada revealed four outlier SNPs (109734_46, 334591_7, 505960_78, and 522238_59) with log_10_(PO) = 1,000 in global comparison associated strongly with environmental variables: CC with aspect and TT with number of rainfall days per year for SNP 109734_46 (the minor C allele frequencies were 0.92, 0.86, 0.79, 0.20, and 0.00, respectively, for populations JGL, GPL, ST, DW30, and DW41), TT and GG with annual mean temperature for SNP 334591_7 (the minor T allele frequencies were 0.00, 0.00, 0.00, 0.70, and 1.00, respectively, for populations JGL, GPL, ST, DW30, and DW41), CC and AA with annual mean temperature for SNP 505960_78 (the minor C allele frequencies were 0.00, 0.00, 0.00, 0.71, and 1.00, respectively, for populations JGL, GPL, ST, DW30, and DW41), and GG with aspect and AA with number of rainfall days per year for SNP 522238_59 (the minor G allele frequencies were 0.92, 0.80, 0.64, 0.23, and 0.00, respectively, for populations JGL, GPL, ST, DW30, and DW41). No significant LD between these four outlier SNPs was found with two-locus exact test (Supplementary Table 7). Functional annotation of the sequences containing the outlier SNPs with BLASTN (Supplementary Table 8) found high sequence similarities for locus 227675 (outlier SNP found in global comparison) to the mitochondrial alternative oxidase 1 (AOX1) of *Araucaria angustifolia* (*E* = 5E-10), locus 334591 (outlier SNP found in global and all pairwise population comparisons) to the clone GQ03405_M22 mRNA of *Picea glauca* (*E* = 4E-23), and locus 521876 (outlier SNP found in comparison between the southern neighboring DW30 and DW41 populations) to the mitochondrial large subunit ribosomal RNA gene (LSU rRNA) of *Abies homolepis* (*E* = 3E-35). Clone GQ03405_M22 mRNA sequence of *Pic. glauca* is corresponded with the sequences of mitochondrial cytochrome oxidase subunit 1 (COX1) of *larix gmelinii* (EF053147.1)

**Table 2:** Selective outliers identified by BAYESCAN and FDIST and their correlations with environmental variables analyzed with Samβada.

**SNP ID**	**BAYESCAN log**_**10**_**(PO) in total and pairwise comparisons**								**Significant association of SNP genotypes with environmental variables (Samβada)**
	**5 populations**	**JGL DW30**	**GPL DW30**	**ST DW30**	**JGL DW41**	**GP DW41**	**ST DW41**	**DW30 DW41**	
(1) 63667_37	0.520							0.573^+^	
(2) 93955_78	3.398^+^				0.778^+^	1.987^+^	1,000^+^		AA (RainD)
(3) 109734_33	0.701^+^							0.690^+^	
(4) 109734_46	1,000^+^				3.699^+^	3.398^+^	2.795^+^		CC (Aspect); TT (RainD)
(5) 151653_31								2.467^+^	
(6) 161549_14	1.962^+^								CT (Aspect); TT (Aspect)
(7) 207023_57	3.097^+^				2.191^+^	1.392^+^	1.570^+^		AA (RainD)
(8) 227675_81	1.186^+^								
(9) 280158_34		0.625^+^		0.644^+^					CC (Soil pH)
(10) 313537_25	1.307^+^		0.517^+^			1.425^+^			TT (RainD, Aspect)
(11) 315865_72		0.556^+^		0.630^+^					
(12) 334591_7	1,000^+^	1.144^+^	1.055^+^	1.149^+^	1,000^+^	1,000^+^	1,000^+^		TT (BIO1); GG (BIO1)
(13) 340782_17	2.104^+^								AA (BIO12, RainD); AG (BIO12, RainD)
(14) 341940_10								1.352^+^	
(15) 341940_78							1.086^+^		
(16) 505960_78	1,000^+^	1.483^+^	0.807^+^	1.400	1,000^+^	1,000^+^	1,000^+^		AA (BIO1); CC(BIO1)
(17) 521876_50								1.528^+^	TT (BIO1); GT (BIO1)
(18) 521876_51								1.525^+^	CC (BIO1); AC (BIO1)
(19) 522238_59	1,000^+^				1,000^+^	2.234^+^	1.507^+^		GG (Aspect); AA (RainD)
(20) 559821_24	1.553^+^								
(21) 638724_65	1.240^+^								AA (BIO1); AC (BIO1)
(22) 638724_71	1.294^+^								GG (BIO1); AG (BIO1)
(23) 734440_39	3.398^+^	0.917^+^			0.650^+^				AA (Aspect)

*BIO1, annual mean temperature; BIO12, annual precipitation; RainD, number of rainfall days per year*.

+ *Represents outliers also detected by FDIST*.

*In Samβada analysis, significant association between SNP genotypes and environmental variables was determined using false discovery rate of 1% in 483 comparisons*.

### Genetic differentiation

Both AMOVA and *F*_ST_ measures revealed significant differentiation between *K. davidiana* and *K. davidiana* var. *formosana* (*Φ*_CT_ = 0.233, *P* = 0.001; *F*_ST_ = 0.077, *P* = 0.001; Table 3). The levels of genetic differentiation were shallow but significant in all comparisons when the total and neutral data sets were used in the analyses. However, significantly high levels of genetic differentiation were found in all comparisons using the outlier data set (Table 3). Across populations of *K. davidiana* var. *formosana*, significant *Φ*_ST_ (= 0.422, *P* = 0.001) and across population *F*_ST_ (= 0.427, *P* = 0.001) were found based on the outlier data. Comparing between populations of the North and South regions, *Φ*_ST_ was 0.425 (*P* = 0.001) and *F*_ST_ was 0.437 (*P* = 0.001). Significant AMOVA and *F*_ST_ were also found between populations within the North (*Φ*_ST_ = 0.133, *P* = 0.001; *F*_ST_ = 0.125, *P* = 0.001) and within the South (*Φ*_ST_ = 0.326, *P* = 0.001; *F*_ST_ = 0.290, *P* = 0.001).

**Table 3:** Summary of the analysis of molecular variance (AMOVA) and across population *F*_ST_.

	**Genetic differentiation**		
**Source of variation**	**Total data**	**Neutral data**	**Outlier data**
Between species	*Φ*_CT_ = 0.233 (0.001) *F*_ST_ = 0.077 (0.001)		
Between populations of KDF	*Φ*_ST_ = 0.0775 (0.001) *F*_ST_ = 0.022 (0.002)	*Φ*_ST_ = 0.074 (0.001) *F*_ST_ = 0.019 (0.002)	*Φ*_ST_ = 0.422 (0.001 *F*_ST_ = 0.427 (0.001)
Between northern and southern populations of KDF	*Φ*_ST_ = 0.061 (0.001) *F*_ST_ = 0.010 (0.001)	*Φ*_ST_ = 0.056 (0.001) *F*_ST_ = 0.016 (0.001)	*Φ*_ST_ = 0.425 (0.001) *F*_ST_ = 0.437 (0.001)
Between northern populations of KDF	*Φ*_ST_ = 0.032 (0.001) *F*_ST_ = 0.005 (0.003)	*Φ*_ST_ = 0.034 (0.001) *F*_ST_ = 0.005 (0.200)	*Φ*_ST_ = 0.133 (0.005) *F*_ST_ = 0.125 (0.002)
Between southern populations of KDF	*Φ*_ST_ = 0.067 (0.001) *F*_ST_ = 0.018 (0.001)	*Φ*_ST_ = 0.065 (0.001) *F*_ST_ = 0.016 (0.001)	*Φ*_ST_ = 0.326 (0.001) *F*_ST_ = 0.290 (0.001)

*Results represent comparison between Taiwan cow-tail fir and its ancestor, Keteleeria davidiana and comparisons between populations of Taiwan cow-tail fir under different scenarios. Values within parentheses are P-values*.

### Genetic clustering

**Figure 2:**
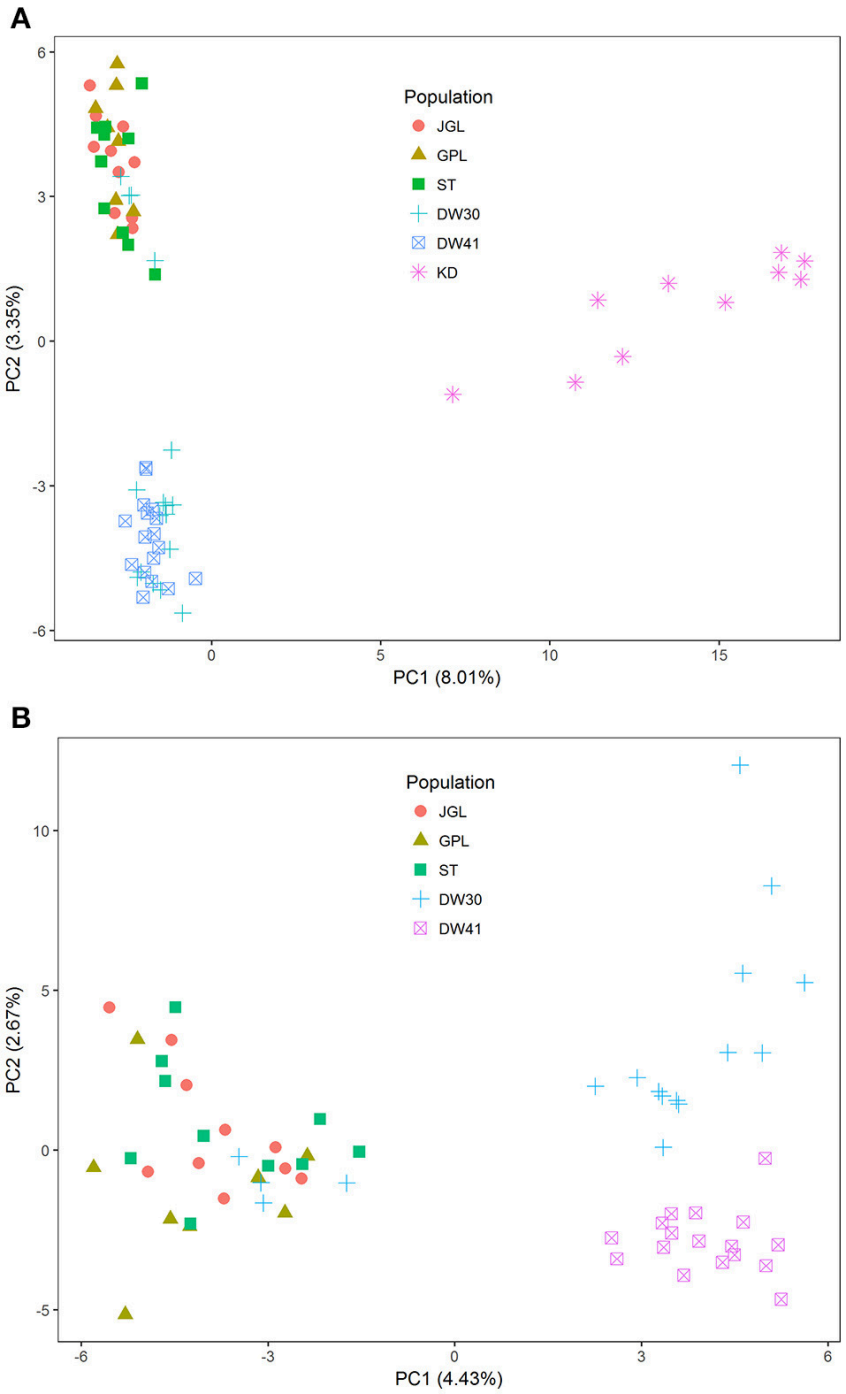
Scatter plots of the first two principal components (PCs) based on allelic frequencies of SNPs. **(A)** Samples of Taiwan cow-tail fir and *Keteleeria davidiana* (*n* = 72) and **(B)** samples of Taiwan cow-tail fir (*n* = 62). See Table 1 for population code abbreviations for Taiwan cow-tail fir. KD, *K. davidiana*.

Using the total data, eigenvalues for the first two PCs were 33.43 and 14.00 and 16.32 and 9.84, when both species and *K. davidiana* var. *formosana* only were analyzed, respectively. However, only small amounts of genetic variation were explained by the first two PCs (both species: PC1 = 8.01%, PC2 = 3.35%; *K. davidiana* var. *formosana* only: PC1 = 4.43%, PC2 = 2.67%) (Figure 2), suggesting that only minor proportion of SNPs possessed the power of species delimitation and individual distinction, and most alleles were shared between ancestor and derivative species (95.0 ± 1.35%) and between populations of *K. davidiana* var. *formosana* (94.7 ± 0.4%), but effective genetic clustering was observed. PCA revealed clear distinction between *K. davidiana* and *K. davidiana* var. *formosana* (Figure 2A). In general, three distinct clusters of the northern, southern DW30, and southern DW41 populations of *K. davidiana* var. *formosana* were found (Figure 2B), however, with amalgamation of four DW30 individuals with the northern cluster. Individuals of the DW41 population were found to be distinct genetically.

**Figure 3:**
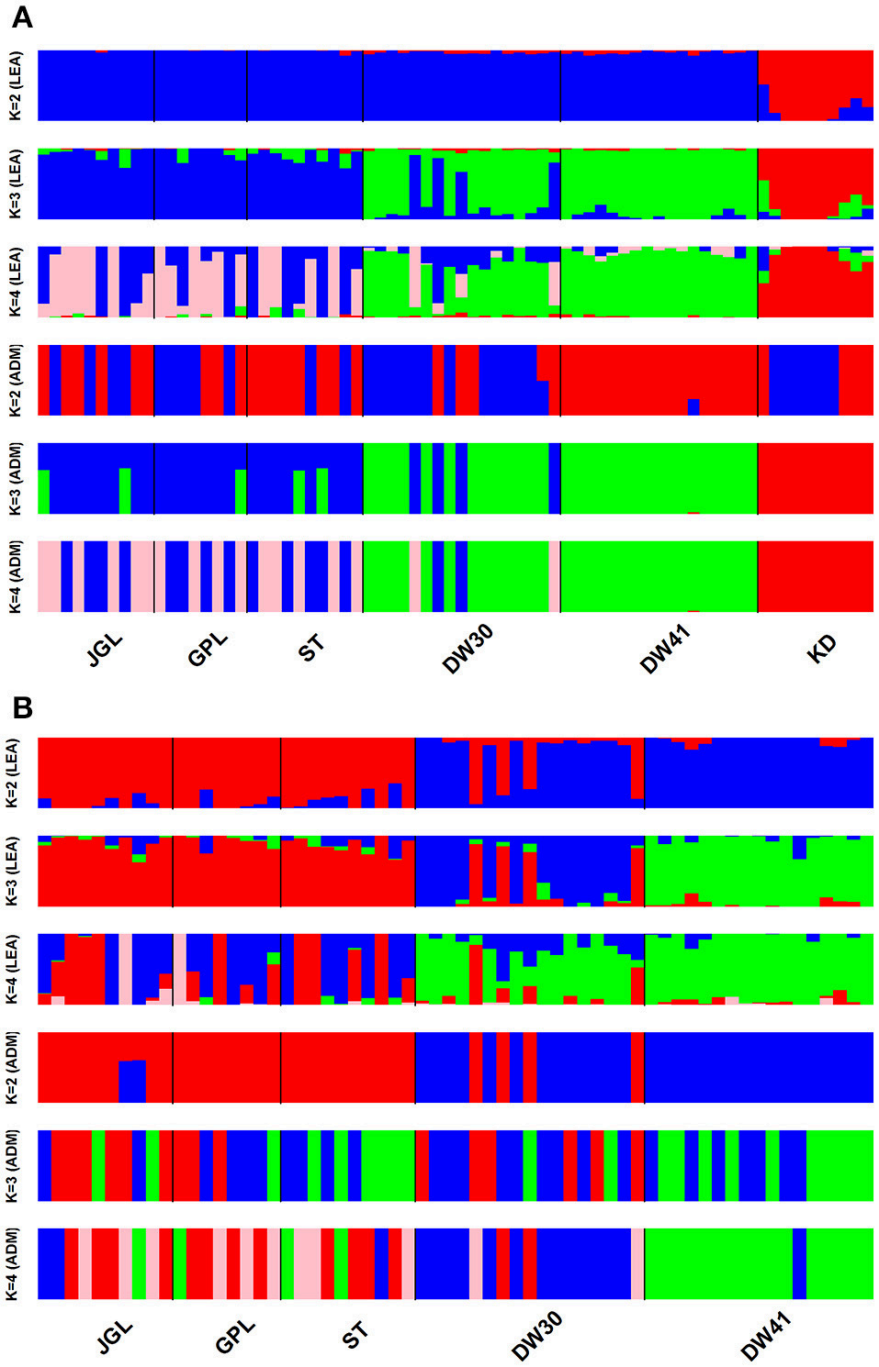
Barplots represent inference of individual assignments based on LEA and ADMIXTURE (ADM) for different clustering scenarios (*K*). **(A)** Samples of Taiwan cow-tail fir and *Keteleeria davidiana* (*n* = 72) and **(B)** samples of Taiwan cow-tail fir (*n* = 62). See Table 1 for population code abbreviations. KD, *K. davidiana*.

**Figure 4:**
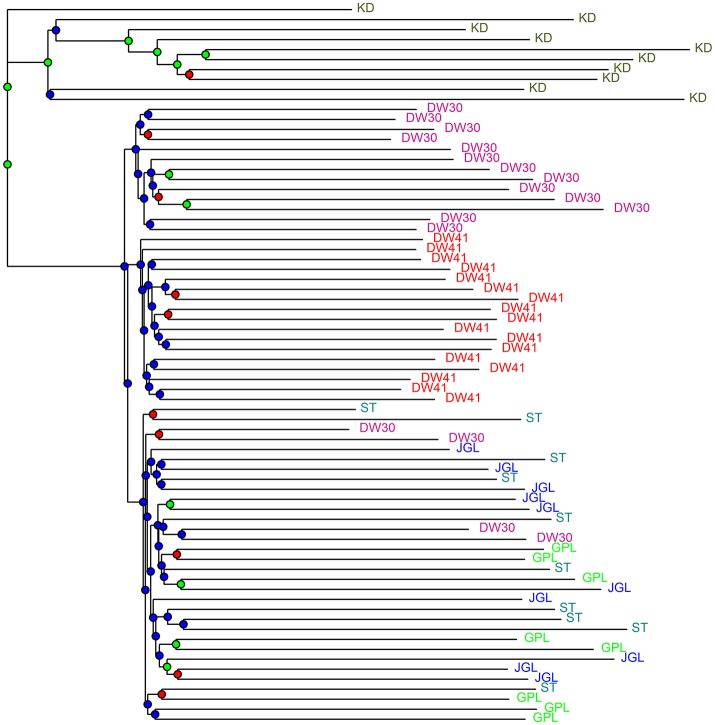
Neighbor-joining tree of samples of Taiwan cow-tail fir and *Keteleeria davidiana* based on Nei's genetic distance. Bootstrap support values (BSVs) were coded with colored nodes with BSVs ≥ 90% (green), 90% < BSVs ≥ 70% (red), and BSVs < 70% (blue), respectively. See Table 1 for population code abbreviations. KD, *K. davidiana*.

CE was minimized at *K* = 2 and *K* = 1, respectively, when data included *K. davidiana* and *K. davidiana* var. *formosana* and data included only *K. davidiana* var. *formosana*, based on sNMF algorithm of LEA (Supplementary Figures 3A,B). Individual ancestry inferred with ADMIXTURE found CV error was minimized at *K* = 1 in both data sets, and no difference for 10 runs of a given *K* was found estimating CV (Supplementary Figures 3C,D). However, genetically homogeneous groups that resolved substructure with the highest biological meaning revealed otherwise. With LEA and ADMIXTURE, a clear phylogenetic break between *K. davidiana* and *K. davidiana* var. *formosana* was observed at *K* = 3 and 4 based on the total data (Figure 3A). Both LEA and ADMIXTURE showed distinction between northern and southern populations of *K. davidiana* var. *formosana* when *K* = 4 (Figures 3A,B). Admixtures between individuals of the southern DW30 population and northern populations were also observed. Individuals of the southern DW41 population were clearly separated from individuals of all other populations of *K. davidiana* var. *formosana* at *K* = 3 analyzed with LEA and at *K* = 4 analyzed with ADMIXTURE (Figure 3B). Similar pattern of genetic structuring was also found based on the NJ tree (Figure 4). The NJ tree revealed a close relationship between *K. davidiana* (KD clade) and the DW30 population of *K. davidiana* var. *formosana* (DW30 clade) forming a KD + DW30 clade, which can be easily collapsed to a KD + DW30 + DW41 clade (BSV > 90%) due to a low BSV of < 70%. The individuals of the three northern populations of *K. davidiana* var. *formosana* formed a well-supported separate clade with a BSV of >90%.

### Importance of environmental variables explaining genetic variation and the effect of environment and geography on genetic variation of taiwan cow-tail fir

Because similar results were found for the forward selection and MMRR analyses based on the total and neutral data sets, only the results based on the total and outlier data sets are reported (Tables 4, 5). All environmental variables explained little genetic variation but was significant (*F*-test) when the total data was used (Table 4). When the outlier data was used in the forward selection, substantial amounts of outlier genetic variation were explained significantly by environmental variables. The most important environmental variables that explained outlier genetic variation were number of rainfall days per year (adjusted *R*^2^ = 0.311, *F* = 28.55, *P* = 0.001), aspect (adjusted *R*^2^ = 0.230, *F* = 19.18, *P* = 0.001), and annual mean temperature (adjusted *R*^2^ = 0.164, *F* = 12.95, *P* = 0.001). Soil pH (adjusted *R*^2^ = 0.090, *F* = 9.99, *P* = 0.001), annual precipitation (adjusted *R*^2^ = 0.070, *F* = 6.49, *P* = 0.001), and slope (adjusted *R*^2^ = 0.041, *F* = 4.33, *P* = 0.001) were also found to significantly explain the outlier genetic variation.

**Table 4:** Forward selection of environmental variables classified into three categories (bioclimate, ecology, and topology) explaining genetic variation within Taiwan cow-tail fir.

	**Environmental variables**	**Total**			**Outlier**		
		***R*^2^**	**Adjusted *R*^2^**	***F* (*P*)**	***R*^2^**	**Adjusted *R*^2^**	***F* (*P*)**
Bioclimate	BIO1BIO12	0.029 0.023	0.012 0.007	1.762 (0.001) 1.411 (0.001)	0.177 0.081	0.164 0.070	12.95 (0.001) 6.49 (0.001)
Ecology	RainD Soil pH NDVI	0.037 0.024 0.019	0.021 0.008 0.003	2.329 (0.001) 1.490 (0.001) 1.174 (0.001)	0.322 0.098	0.311 0.090	28.55 (0.001) 9.99 (0.001)
Topology	Aspect Slope	0.003 0.002	0.017 0.005	2.046 (0.001) 1.290 (0.001)	0.242 0.052	0.230 0.041	19.18 (0.001) 4.33 (0.001)

*BIO1, Annual mean temperature; BIO12, annual precipitation; RainD, number of rainfall days per year; NDVI, normalized difference vegetation index*.

**Table 5:** Results of multiple matrix regression with randomization (MMRR) analysis.

	***R*^2^**	**β_D_ (*P*)**	**β_E_ (*P*)**
**TOTAL**			
Genetic vs. environment	0.013		0.318 (0.553)
Genetics vs. geography	0.303	0.181 (0.052)	
Genetic vs. environment + geography	0.329	0.209 (0.117)	−0.059 (0.538)
**OUTLIER**			
Genetic vs. environment	0.822		0.924 (0.045)
Genetics vs. geography	0.904	0.904 (0.017)	
Genetic vs. environment + geography	0.974	0.591 (0.064)	0.430 (0.040)

*MMRR analysis inferring the effects of geographic (β_D_) and environmental (β_E_) distances based on Nei's genetic distance (Nei, 1978) using the total and outlier data. R^2^ represents the total amount of genetic variation explained by either geography or environment, or the combined effect of geography and environment*.

Strong correlation between environmental and geographic distance matrices was found (Mantel *r* = 0.465, *P* = 0.025). Essentially no correlation was found between genetic variation and environment, between genetic variation and geography, and between genetic variation and combined effect of environment and geography, based on the total data (Table 5). Using the outlier data, significant IBE was found between genetic variation and environment (*R*^2^ = 0.822, β_E_ = 0.924, *P* = 0.045). Significant IBD (between genetic variation and geography) was also found (*R*^2^ = 0.904, β_D_ = 0.904, *P* = 0.017). When considering both environment and geography, results showed a pattern of strong IBE based on the outlier data (*R*^2^ = 0.974; IBD: β_D_ = 0.591, *P* = 0.064; IBE: β_E_ = 0.430, *P* = 0.040).

## Discussion

### Species delimitation between *K. davidiana* and *K. davidiana* var. *formosana*

Species delimitation using genome-wide markers has been demonstrated in plants (e.g., Eaton and Ree, 2013; Paun et al., 2016). In the present study, AMOVA showed significantly high differentiation at species level (*Φ*_CT_ = 0.233, *P* = 0.001) though between species *F*_ST_ was low but also significant (*F*_ST_ = 0.077, *P* = 0.001). Nevertheless, distinction between the closely related island and mainland *Keteleeria* species pair were elucidated using ddRADseq. Three lines of evidence, including PCA (Figure 2A), individual assignments (LEA and ADMIXTURE, Figure 3A), and the NJ tree (Figure 4), suggest that each of species studied is distinct. Although the NJ tree revealed that *K. davidiana* (KD clade) was most closely related to the DW30 population of *K. davidiana* var. *formosana, Keteleeria* colonization from China into southern Taiwan cannot be inferred based solely on the NJ tree. Moreover, the disappearance of *K. davidiana* var. *formosana* from its historically occupied habitats in central Taiwan (Tsukada, 1967) and other areas hinders the investigation of *Keteleeria* colonization route.

### Population genetic diversity and outbreeding of taiwan cow-tail fir

Similar trends in *A*_R_, π, *H*_O_, *H*_E_, and *uH*_E_ were observed across populations of *K. davidiana* var. *formosana* (Supplementary Figure 1). The average levels of population genetic diversity measures, including *A*_R_, π, *H*_O_, *H*_E_, and *uH*_E_ (Table 1), were lower in restricted and disjunctly distributed *K. davidiana* var. *formosana* compared with widespread species of Pinaceae, e.g., *Pic. glauca* (white spruce) (*A*_R_ = 1.920, *H*_O_ = 0.276, *uH*_E_ = 0.270, Namroud et al., 2008), *Pic. mariana* (black spruce) (*A*_R_ = 1.850, *H*_O_ = 0.241, *H*_E_ = 0.247, Prunier et al., 2011), *Pinus albicaulis* (whitebark pine) (*A*_R_ = 1.93, *H*_O_ = 0.32, *H*_E_ = 0.35, *uH*_E_ = 0.36, Liu et al., 2016), and *Pic. abies* (Norway spruce) (π = 0.0893, *H*_O_ = 0.138, *H*_E_ = 0.258, Fagernäs, 2017). In the present study, we found that populations of *K. davidiana* var. *formosana* appear to harbor substantial amount of variation relative to *K. davidiana* (*A*_R_: 98.3%, π: 80.0%, *H*_O_: 82.4%, *H*_E_: 81.1%, and *uH*_E_: 80.0%) and may have potential for adaptive evolution under natural selection (Petit and Hampe, 2006; Barrett and Schluter, 2008).

Within *K. davidiana* var. *formosana*, the northern populations had comparatively higher levels of genetic diversity measures than those in the southern populations (Table 1). The levels of *H*_E_ were lower than the level of *H*_O_ in all populations, resulting in negative *F*_IS_-values. A negative value of *F*_IS_ indicates heterozygote excess and is typical of conifers that are outcrossing (Hamrick et al., 1992; Hamrick and Godt, 1996). An excess of heterozygosity could indicate negative assortative mating or higher fitness of heterozygotes (Lachance, 2008). Moreover, significant non-zero *I*_A_ and *r*_D_ values were only found in the DW41 population, indicating significant non-random associations between alleles, which might have related to the higher degree of local adaptation in the DW41 than in other populations of Taiwan cow-tail fir (Bürger and Akerman, 2011). However, all *K. davidiana* var. *formosana* populations showed significant non-zero *I*_A_ and *r*_D_ values based on AFLP (Fang et al., 2013). The discrepancy could be due to the probable excess of homozygous genotypes resulted from the loss of restriction sites in AFLP, however, the presence/absence of restriction sites is not the primary source of information in ddRADseq (Cariou et al., 2016). We used the same restriction enzymes in the present study as that used in the AFLP study, standard errors of the estimates of all genetic diversity measures in all populations were similar and smaller based on ddRADseq, while dissimilar and larger standard errors of population *uH*_E_ were observed based on AFLP (Fang et al., 2013), suggesting more reliable estimation of *I*_A_ and *r*_D_ within populations based on ddRADseq data. It is likely that errors produced during the PCR and sequencing can be filtered away and producing a large number of reliable high quality SNPs for population genetic analysis.

Multilocus LD based on ddRADseq revealed only one population (DW41) had significant non-zero *I*_A_ and *r*_D_ values, indicating rapid decay of LD over time in most examined populations of *K. davidiana* var. *formosana* conforming to studies of other Pinaceae species (Brown et al., 2004; Neale and Savolainen, 2004; Heuertz et al., 2006). Retention of significant LD in the DW41 population may have been caused by recent bottlenecks (Petit and Hampe, 2006) and/or natural selection (Barrett and Schluter, 2008; Eckert et al., 2010). Nonetheless, we cannot exclude the possibility of past bottlenecks occurred in all populations since the levels of genetic diversity measures were low across populations compared with widespread Pinaceae species (Namroud et al., 2008; Prunier et al., 2011; Liu et al., 2016; Fagernäs, 2017).

### Population differentiation and structure of taiwan cow-tail fir

The levels of genetic differentiation within *K. davidiana* var. *formosana* based on the total and neutral data sets are in accordance with the general realization that conifers typically exhibit low population differentiation due to long distance wind pollination (Hamrick et al., 1992; Hamrick and Godt, 1996). Based on the total data, significant differentiation between *K. davidiana* and *K. davidiana* var. *formosana* was found (Table 3), suggesting apparent distinction between ancestor-derivative species pair. Only moderate level of genetic differentiation was found when compared between northern populations based on the outlier data (Table 3), and we did not detect any outlier SNPs when compared between the northern populations based on BAYESCAN and FDIST. High levels of genetic differentiation were found when comparisons involved the southern DW30 and DW41 populations based on the outlier data. These results are only partially concordant to the results of previous AFLP study (Fang et al., 2013), in which moderate level of differentiation based on *F*_ST_ outliers was found, probably because of high level of stringency applied in the identification of *F*_ST_ outliers in the present study.

Genetic clustering, based on the total data, using ddRADseq data in the present study provided a prominent phylogeographic break between northern and southern populations within *K. davidiana* var. *formosana* compared with no clear northern-southern distinction based on the total AFLP data (Fang et al., 2013). The admixtures of individuals between the southern DW30 population and northern populations, based on PCA (Figure 2B), LEA (Figure 3), ADMIXTURE (Figure 3), and the NJ tree (Figure 4), might have resulted from incomplete lineage sorting of ancestral variation or recent hybridization. Long distance seed dispersal between the southern DW30 population and northern populations may be less likely because of the recalcitrant seed storage behavior (Yang et al., 2006) and low rate of regeneration in natural stands (Wang, 1987) in *K. davidiana* var. *formosana*. Moreover, the NJ tree showed close relationship between *K. davidiana* and the southern populations of *K. davidiana* var. *formosana*. This result suggests that recent hybridization between the southern DW30 population and northern populations could be probable via effective pollen dispersal, in agreement with the ages of old growth trees between 100 and 300 years. Viable long distance pollen migration in *Pin. taeda* of at least 41 km was found (Williams, 2010). Pine pollens can travel up to 600–1,000 km (Dyakowska, 1948), whereas later Dyakowska (1959) suggested a pollen traveling range of 74.7 km. Szczepanek et al. (2017) reported a pine pollen travel distance of 500–750 km from Ukraine and Slovakia to southern Poland. In addition, viable long distance pollen dispersal can be aided by tropical cyclones and seasonal monsoons (Williams, 2009). Hence, recent hybridization between the southern DW30 population and northern populations of *K. davidiana* var. *formosana* via effective pollen migration could be possible.

Factors such as spatial context of selection and the balance between the strength of divergent selection and the between-population migration rates are important influencing population divergence (Endler, 1973; Lenormand, 2002). Individuals of the three northern populations were not clearly distinguished from each other probably because of the high rate of effective pollen dispersal among populations that are in geographical proximity, particularly in wind-pollinated conifers (Hamrick et al., 1992; Hamrick and Godt, 1996). Spatial environmental heterogeneity at overall scale among *K. davidiana* var. *formosana* populations was suggested by significant Mantel correlation between geographic and environmental distances (Mantel *r* = 0.465, *P* = 0.025). Spatial environmental heterogeneity and strong IBE based on the outlier data (Table 5) suggest that gene flow, particularly between the two southern neighboring populations (DW30 and DW41), could have been restricted due probably to habitat isolation or immigrant inviability arisen from local optimal for the environment and reduced survival and reproduction of migrants (Nosil et al., 2005; Jump and Peñuelas, 2006).

### Adaptive divergence in association with environmental heterogeneity in taiwan cow-tail fir despite significant role of geography on population differentiation

Evolutionary theory predicts that population genetic divergence should be correlated with both geographic distance and environmental heterogeneity. Gene flow among populations could follow both IBD and IBE patterns, corresponding to geographic distance and environmental context (Sexton et al., 2014). Of the 26 studies related to plants summarized by Sexton et al. (2014), 38.5% found both IBD and IBE patterns, and 30.8 and 11.5% found IBD and IBE, respectively. In the present study, geographic distance was strongly correlated with environmental distance (Mantel *r* = 0.465, *P* = 0.025) suggesting that both IBD and IBE may have influenced population divergence within *K. davidiana* var. *formosana*. No significant correlation was found between genetic and environmental distances and between genetic and combined effect of environmental and geographic distances based on the total data using MMRR (Table 5). However, marginal significance between genetic and geographic distances (β_D_ = 0.181, *P* = 0.052) was found based on the total data, suggesting that IBD could have played an important role in shaping the population genetic divergence of Taiwan cow-tail fir. When the outlier data was used, genetic variation was significantly correlated with either geographic or environmental (β_D_ = 0.904, *P* = 0.017; β_E_ = 0.924, *P* = 0.045) distances. In addition, strong IBE was found when considering combined effect of geography and environment (β_D_ = 0.591, *P* = 0.064; β_E_ = 0.430, *P* = 0.040). Our results suggest adaptive divergence corresponding to environmental heterogeneity despite strong IBD within *K. davidiana* var. *formosana*.

With the seven environmental variables retained that were separated into three categories: bioclimate, ecology, and topology, annual mean temperature, number of rainfall days per year, and aspect were found to be the most important environmental factors that explained substantial amounts of the outlier genetic variation using forward selection (Table 4). Results of forward selection are conformed to the results of Samβada analysis that 15 outlier SNPs were found to be strongly correlated with environmental variables using multiple univariate logistic regression (Table 2). Environmental gradients of annual temperature, number of rainfall days per year, and aspect are the most important environmental factors influencing adaptive variation in *K. davidiana* var. *formosana*. However, the second most important environmental factors, including annual precipitation, soil pH, and slope could also be important environmental factors that may have played key roles in driving adaptive divergence in *K. davidiana* var. *formosana*.

Temperature and precipitation are commonly found to play prominent roles as selective drivers for adaptive variation in various plant species (Manel et al., 2010, 2012; Bothwell et al., 2013; Fang et al., 2013; Hsieh et al., 2013; Huang et al., 2015). The topographic factor aspect is an important predictor of forest attributed to differences in radiation exposure and has a strong influence on the microclimate (Rosenberg et al., 1983; Bennie et al., 2008), and was found to be associated with genetic variation within (Manel et al., 2010, 2012; Bothwell et al., 2013) and between species (Nakazato et al., 2010; Huang et al., 2015). Slope is also a factor that may influence habitat microclimate (Brousseau et al., 2015) and contributed to intra- and inter-species adaptive divergence (Monahan et al., 2012; Brousseau et al., 2015). Small-scale habitat variation in soil alkali content has been found to be involved in intraspecific adaptive divergence of photosynthetic traits in a grass species, *Phragmites australis* (Qiu et al., 2017). In addition, Pease et al. (2016) found non-synonymous mutations in 43 genes strongly associated with soil pH in rapidly diverged wild *Solanum* species. In *K. davidiana* var. *formosana*, the aspect of the southern DW30 (34.7°) and DW41 (61.5°) (Supplementary Table 2) populations facing northeast, thereby, causing periodical drier conditions implying water stress or desiccation due to foehn winds induced by tropical cyclones (Chen et al., 2010). Tropical cyclones and the seasonal monsoon rainfall may have dramatically raised the amount of precipitation (Chen and Chen, 2011) in the southern DW41 population that had the highest annual precipitation (4,810 mm/year) compared with other populations (Supplementary Table 2). The more alkali condition of the southern DW30 population (soil pH = 5.5) compared with other populations may have played an important role in the divergence with the neighboring DW41 population. Therefore, the large-scale (temperature, number of rainfall days per year, precipitation) and the small-scale (aspect, slope, and soil properties) habitat variations could have played critical roles in shaping the population divergence between geographically distant and neighboring populations of *K. davidiana* var. *formosana*.

Sequences flanking three outlier SNPs that associated strongly with environmental variables were found to have high sequence similarities with low *E*-values to specific genes of mitochondrial AOX, COX, and LSU rRNA based on BLASTN search (Supplementary Table 8). The finding of the three annotated outlier SNPs associated with mitochondrial genes is interesting because mitochondrial genome is maternally inherited in Pinaceae (Hipkins et al., 1994) and displays higher subdivision among populations than paternally or biparentally inherited genes (Petit et al., 2005), and mitochondrial genes are known to play critical roles in plant local adaptation (Bock et al., 2014; Sloan, 2015). At the end of mitochondrial electron transport chain, oxygen can be reduced to water by either COX or AOX (Millar et al., 2008; Kühn et al., 2015). AOX and COX can relax the highly coupled and tensed electron transport process of mitochondria hence providing and maintaining metabolic homeostasis by reducing oxygen to water (Vanlerberghe, 2013). AOX and COX are also found to be important in stress signaling and plant stress response (Bartoli et al., 2005; Vacca et al., 2006; Costa et al., 2007; Dahan et al., 2014; Kühn et al., 2015). Mitochondrial ribosomal RNA genes were found to be involved in robustness of cell growth, proliferation, and therefore the whole plant survival (Greber et al., 2014). Our results suggest that these three outlier SNPs potentially evolved under selection may have involved in the growth and survival of locally adapted lineages in *K. davidiana* var. *formosana*.

## Conclusions

Genome-wide SNPs obtained using ddRADseq has permitted for the evaluation of species delimitation between *K. davidiana* and *K. davidiana* var. *formosana*, and assessment of genetic diversity and fine-scale population genetic structure in *K. davidiana* var. *formosana*. Unlike AFLP data (Fang et al., 2013), genome-wide SNPs provided distinct regional substructuring within *K. davidiana* var. *formosana* based on the total data. Significant negative *F*_IS_ values estimated with SNP data in the present study reflected outbreeding of *K. davidiana* var. *formosana* typical of conifers. The present study highlights the separation of environmental variables into separate categories for investigation of the impact on genetic variation. We identified outlier SNPs potentially evolved under selection strongly associated with specific environmental variables that might have played important roles in maintaining metabolic homeostasis and survival underlying local adaptation despite significant IBD. The present study emphasizes the importance of examining population isolation using ddRADseq to understand the spatial distribution of genetic variation across a species range. Our analyses suggest adaptive divergence between allopatrically distributed northern and southern populations and between the geographically neighboring DW30 and DW41 populations attributed to environmental heterogeneity. In addition, differential contributions of seed dispersal and pollen migration might have played crucial roles in shaping the population structure and spatial distribution of genetic diversity across species range of Taiwan cow-tail fir.

## Author contributions

S-YH proposed, funded, and designed the research; J-DC, K-MS, S-YH, and Y-CC collected samples; K-MS performed research; S-YH, K-MS, C-TC, and J-DC analyzed data; S-YH and K-MS wrote the paper. All authors have read and approved the final manuscript.

### Conflict of interest statement

The authors declare that the research was conducted in the absence of any commercial or financial relationships that could be construed as a potential conflict of interest.

## Supplementary material

Supplementary Table 1 Determination of the values of parameters in the discovery of SNPs in STACKS pipeline.

Supplementary Table 2 Site environmental variables, including site names, annual mean temperature (BIO1), annual precipitation (BIO12), number of rainfall days per year (RainD), normalized difference vegetation index (NDVI), Soil pH, aspect (0–360°), and slope (0–90°), of the five populations of Taiwan cow-tail fir.

Supplementary Table 3 Number of SNP in data sets containing non-missing genotypes in at least 40, 50, and 60% of samples across populations.

Supplementary Table 4 Overview of STACKS pipeline analyses for ddRADseq.

Supplementary Table 5 *P*-values of pairwise Kolmogorov Smirnov test for distributions of genetic diversity measures across populations and pairwise locus *F*_ST_ between data sets of non-missing genotypes in at least 40, 50, and 60% of samples across populations.

Supplementary Table 6 Summary of population genetic parameters in *Keteleeria davidiana* and Taiwan cow-tail fir based on ddRADseq. KD, *K. davidiana*.

Supplementary Table 7 Test of two-locus linkage disequilibrium between the outlier SNPs identified by *F*_ST_-based methods.

Supplementary Table 8 Summary of the sequences containing outlier SNPs matches to GenBank gene sequences using BLASTN.

Supplementary Figure 1 Distributions of population genetic diversity measures, including allelic richness, nucleotide diversity, observed heterozygosity, expected heterozygosity, and unbiased expected heterozygosity in data sets of non-missing genotypes in at least 40, 50, and 60% of samples across populations.

Supplementary Figure 2 Distributions of pairwise locus *F*_ST_ in data sets of non-missing genotypes in at least 40, 50, and 60% of samples across populations.

Supplementary Figure 3 Minimal cross-entropy and cross validation error analyzed using LEA and ADMIXTURE. **(A,B)** LEA and **(C,D)** ADMIXTURE using samples included both Taiwan cow-tail fir (KDF) and *Keteleeria davidiana* (KD) or samples of Taiwan cow-tail fir.

Supplementary File 1 A php script for the addition of a 5-bp C and a 5-bp T, respectively, to the end of PE read 1 and read 2.

Data Sheet 1 Fifty percent missing data set for the five populations of *Keteleeria davidiana* var. *formosana* in STRUCTUR format (Pritchard et al., 2000).

Data Sheet 2 Fifty percent missing data set for samples include *Keteleeria davidiana* and *K. davidiana* var. *formosana* in STRUCTUR format (Pritchard et al., 2000).
