# Supplementary material for: Adaptive Genetic Divergence Despite Significant Isolation-by-Distance in Populations of Taiwan Cow-Tail Fir (Keteleeria davidiana var. formosana)
Source: Front Plant Sci. 2018 Feb 1;9:92. doi: 10.3389/fpls.2018.00092 (PMC5799944; doi:10.3389/fpls.2018.00092)
Supplement: Supplementary Table 1 — Determination of the values of parameters in the discovery of SNPs in STACKS pipeline. [file Table1.DOCX]

**Supplementary Table 1| Determination of the values of parameters in the discovery of SNPs in STACKS pipeline.**

| Number of SNP obtained when SNP was found in two of the three individuals of the 18 samples used in determination of parameters m, M, and n. | | | | | | |
| --- | --- | --- | --- | --- | --- | --- |
|  | 0 | 1 | 2 | 3 | 4 | 5 |
| m |  |  |  | 5270 | 3515 | 2917 |
| M | 738 | 3279 | 5279 | 5283 | 5769 | 2819 |
| n | 5853 | 5270 | 3796 | 3129 | 2967 | 2819 |
| Percentage of polymorphic loci for different values of parameter m when parameters M and n were set to 2 and 1, respectively. | | | | | | |
| m |  |  | 3 | 4 | 5 |  |
|  |  |  | 0.50% | 0.52% | 0.59% |  |
| Percentage of polymorphic loci for different values of parameter M when parameters m and n were set to 3 and 1, respectively. | | | | | | |
| M | 0 | 1 | 2 | 3 | 4 | 5 |
|  | 0.08% | 0.30% | 0.50% | 0.50% | 0.58% | 0.36% |
