# Supplementary material for: Adaptive Genetic Divergence Despite Significant Isolation-by-Distance in Populations of Taiwan Cow-Tail Fir (Keteleeria davidiana var. formosana)
Source: Front Plant Sci. 2018 Feb 1;9:92. doi: 10.3389/fpls.2018.00092 (PMC5799944; doi:10.3389/fpls.2018.00092)
Supplement: Supplementary Table 2 — Site environmental variables, including site names, annual mean temperature (BIO1), annual precipitation (BIO12), number of rainfall days per year (RainD), normalized difference vegetation index (NDVI), Soil pH, aspect (0–360°), and slope (0–90°), of the five populations of Taiwan cow-tail fir. [file Table2.DOCX]

**Supplementary Table 2| Site environmental variables, including site names, annual mean temperature (BIO1), annual precipitation (BIO12), number of rainfall days per year (RainD), normalized difference vegetation index (NDVI), Soil pH, aspect (0‒360º**)**, and slope (0‒90º), of the five populations of Taiwan cow-tail fir*.***

| Population | BIO1 | BIO12 | RainD | SoilpH | NDVI | Aspect | Slope |
| --- | --- | --- | --- | --- | --- | --- | --- |
| JGL | 208 | 3517 | 183.61 | 4.7 | 0.810 | 316.8 | 35.8 |
| GPL | 192 | 3521 | 184.66 | 4.8 | 0.865 | 273.1 | 24.9 |
| ST | 192 | 3532 | 185.41 | 4.7 | 0.870 | 131.7 | 34.3 |
| DW30 | 197 | 3688 | 130.09 | 5.5 | 0.812 | 34.7 | 35.5 |
| DW41 | 179 | 4810 | 134.02 | 4.9 | 0.855 | 61.5 | 31.3 |

Bioclimate data were downloaded from the WorldClim (http://www.worldclim.org/download/) at 30-sec spatial resolution (~ 1 km) (Hijmans et al., 2005). We chose to use the WorldClim v.1.4 data because no difference in the results of the analyses that made use of the two versions of bioclimate data (WorldClim v.1.4 data for 1960‒1990 and WorldClim v.2 for 1970‒2000) was found.

The seven environmental variables were classified into three categories. Bioclimate data downloaded from WorldClim database were classified as bioclimate category. RainD, soil pH, and NDVI were classified as ecology category. Aspect and slope were classified as topology category.
