# Supplementary material for: Adaptive Genetic Divergence Despite Significant Isolation-by-Distance in Populations of Taiwan Cow-Tail Fir (Keteleeria davidiana var. formosana)
Source: Front Plant Sci. 2018 Feb 1;9:92. doi: 10.3389/fpls.2018.00092 (PMC5799944; doi:10.3389/fpls.2018.00092)
Supplement: Supplementary Table 3 — Number of SNP in data sets containing non-missing genotypes in at least 40, 50, and 60% of samples across populations. [file Table3.DOCX]

**Supplementary Table 3| Number of SNP in data sets containing non-missing genotypes in at least 40%, 50%, and 60% of samples across populations.**

| Percentage of missing data across population | 40% | 50% | 60% |
| --- | --- | --- | --- |
| *K. davidiana* + *K. davidiana* var. *formosana* (n =72) | | |  |
| Number of SNPs | 36,284 | 17,982 | 7,396 |
| *K. davidiana* var. *formosana* (n =62) |  |  |  |
| Number of SNPs | 29,316 | 13,914 | 5,321 |
