# Supplementary material for: Adaptive Genetic Divergence Despite Significant Isolation-by-Distance in Populations of Taiwan Cow-Tail Fir (Keteleeria davidiana var. formosana)
Source: Front Plant Sci. 2018 Feb 1;9:92. doi: 10.3389/fpls.2018.00092 (PMC5799944; doi:10.3389/fpls.2018.00092)
Supplement: Supplementary Table 4 — Overview of STACKS pipeline analyses for ddRADseq. [file Table4.DOCX]

**Supplementary Table 4| Overview of STACKS pipeline analyses for ddRADseq.**

| Metric | Number |
| --- | --- |
| Number of raw reads | 582,985,540 |
| Number of retained reads | 574,480,673 |
| Overall number of stacks | 7,663,569 |
| Number of SNP loci retained | 15,094 |
| Average number of stacks per individual | 395,546 |
| Average read depth per stack | 5.29 |
| Range of numbers of stacks | 42,769 ~ 1,525,245 |
| Range of stack depths | 4.7 ~ 7.0 |
