# Supplementary material for: Adaptive Genetic Divergence Despite Significant Isolation-by-Distance in Populations of Taiwan Cow-Tail Fir (Keteleeria davidiana var. formosana)
Source: Front Plant Sci. 2018 Feb 1;9:92. doi: 10.3389/fpls.2018.00092 (PMC5799944; doi:10.3389/fpls.2018.00092)
Supplement: Supplementary Table 5 — P-values of pairwise Kolmogorov Smirnov test for distributions of genetic diversity measures across populations and pairwise locus FST between data sets of non-missing genotypes in at least 40, 50, and 60% of samples across populations. [file Table5.DOCX]

**Supplementary Table 5| *P* values of pairwise Kolmogorov Smirnov test for distributions of genetic diversity measures across populations and pairwise locus *F*_ST_ between data sets of non-missing genotypes in at least 40%, 50%, and 60% of samples across populations.**

|  | *P* value | | |
| --- | --- | --- | --- |
| *A*_R_ |  | 40% | 50% |
|  | 50% | 0.357 |  |
|  | 60% | 0.008 | 0.008 |
| *π* | 50% | 0.357 |  |
|  | 60% | 0.008 | 0.357 |
| *H*_O_ | 50% | 0.357 |  |
|  | 60% | 0.008 | 0.357 |
| *H*_E_ | 50% | 0.082 |  |
|  | 60% | 0.013 | 0.082 |
| *uH*_E_ | 50% | 0.999 |  |
|  | 60% | 0.357 | 0.357 |
| *F*_ST_ | 50% | 0.038 |  |
|  | 60% | 0.006 | 0.405 |
