# Supplementary material for: Adaptive Genetic Divergence Despite Significant Isolation-by-Distance in Populations of Taiwan Cow-Tail Fir (Keteleeria davidiana var. formosana)
Source: Front Plant Sci. 2018 Feb 1;9:92. doi: 10.3389/fpls.2018.00092 (PMC5799944; doi:10.3389/fpls.2018.00092)
Supplement: Supplementary Table 6 — Summary of population genetic parameters in Keteleeria davidiana and Taiwan cow-tail fir based on ddRADseq. KD, K. davidiana. [file Table6.DOCX]

**Supplementary Table 6| Summary of population genetic parameters in *Keteleeria davidiana* and Taiwan cow-tail fir based on ddRADseq.** KD, *K. davidiana*.

| Population | N | *A*_R_ | *π* (SE) | *H*_O_ (SE) | *H*_E_ (SE) | *uH*_E_ | *F*_IS_  (95% CI) |
| --- | --- | --- | --- | --- | --- | --- | --- |
| JGL | 10 | 1.081  (0.001) | 0.084 (0.001) | 0.105 (0.002) | 0.078 (0.001) | 0.084 (0.001) | -0.244*  (- 0.258, - 0.229) |
| GPL | 8 | 1.081 (0.0011) | 0.085 (0.001) | 0.104 (0.002) | 0.077 (0.001) | 0.084 (0.001) | -0.293*  (- 0.258, - 0.229) |
| ST | 10 | 1.081 (0.001) | 0.085 (0.001) | 0.103 (0.002) | 0.078 (0.001) | 0.085 (0.001) | -0.264*  (- 0.278, - 0.2500) |
| DW30 | 17 | 1.074  (0.001) | 0.076 (0.001) | 0.093 (0.002) | 0.072 (0.001) | 0.076 (0.001) | -0.269*  (- 0.282, - 0.256) |
| DW41 | 17 | 1.071  (0.001) | 0.073 (0.001) | 0.093 (0.002) | 0.070 (0.001) | 0.073 (0.001) | -0.226*  (- 0.238, - 0.215) |
| Average |  | 1.078 | 0.080 | 0.100 | 0.075 | 0.080 |  |
| KD | 10 | 1.096  (0.001) | 0.101 (0.001) | 0.121 (0.002) | 0.093 (0.001) | 0.100 (0.001) | -0.243*  (- 0.256, - 0.229) |
