# Supplementary material for: Adaptive Genetic Divergence Despite Significant Isolation-by-Distance in Populations of Taiwan Cow-Tail Fir (Keteleeria davidiana var. formosana)
Source: Front Plant Sci. 2018 Feb 1;9:92. doi: 10.3389/fpls.2018.00092 (PMC5799944; doi:10.3389/fpls.2018.00092)
Supplement: Supplementary Table 7 — Test of two-locus linkage disequilibrium between the outlier SNPs identified by FST-based methods. [file Table7.DOCX]

**Supplementary Table 7| Test of two-locus linkage disequilibrium between the outlier SNPs identified by *F*_ST_-based methods.**

| SNP ID | Significant LD with other outliers |
| --- | --- |
| (1) 63667_37 |  |
| (2) 93955_78 |  |
| (3) 109734_33 | 341940_78; 734440_39 |
| (4) 109734_46 |  |
| (5) 151653_31 | 313537_25; 559821_24 |
| (6) 161549_14 |  |
| (7) 207023_57 |  |
| (8) 227675_81 | 313537_25 (GPL, ST, DW30, DW41); 341940_10; 341940_78 |
| (9) 280158_34 | 313537_25; 315865_72 (DW30, DW41) |
| (10) 313537_25 | 151653_31; 280158_34; 227675_81 (GPL, ST, DW30, DW41) |
| (11) 315865_72 | 280158_34 (DW30, DW41) |
| (12) 334591_7 |  |
| (13) 340782_17 |  |
| (14) 341940_10 | 227675_81; 341940_78 (ST, DW30, DW41); 638724_65; 638724_71 |
| (15) 341940_78 | 109734_33; 341940_10 (ST, DW30, DW41); 227675_81 |
| (16) 505960_78 |  |
| (17) 521876_50 |  |
| (18) 521876_51 |  |
| (19) 522238_59 |  |
| (20) 559821_24 | 151653_31 |
| (21) 638724_65 | 341940_10; 638724_71 |
| (22) 638724_71 | 341940_10; 638724_65 |
| (23) 734440_39 | 109734_33 |
