# Supplementary material for: Adaptive Genetic Divergence Despite Significant Isolation-by-Distance in Populations of Taiwan Cow-Tail Fir (Keteleeria davidiana var. formosana)
Source: Front Plant Sci. 2018 Feb 1;9:92. doi: 10.3389/fpls.2018.00092 (PMC5799944; doi:10.3389/fpls.2018.00092)
Supplement: Supplementary Table 8 — Summary of the sequences containing outlier SNPs matches to GenBank gene sequences using BLASTN. [file Table8.DOCX]

**Supplementary Table 8| Summary of the sequences containing outlier SNPs matches to GenBank gene sequences using BLASTN.**

| Locus ID | Max  score | Total  score | Query  cover | E value | Identity | NCBI sequence ID | Annotation |
| --- | --- | --- | --- | --- | --- | --- | --- |
| 63667 | 42.8 | 42.8 | 33% | 1.5 | 86% | XM_016820816.1 | disease resistance protein RPS2-like *Gossypium hirsutum* |
| 151653 | 46.4 | 46.4 | 25% | 0.12 | 93% | XM_019373747.1 | uncharacterized LOC109210345 [*Nicotiana attenuata*] |
| 227675 | 73.4 | 73.4 | 100% | 5e-10 | 81% | JQ762252.1 | mitochondrial alternative oxidase 1 (AOX1) gene, exon 3 and partial cds [ *Araucaria angustifolia*] |
| 280158 | 41 | 41 | 29% | 89 | 93% | XM_007201984.2 | lycopene beta cyclase, chloroplastic/chromoplastic  [*Prunus persica*] |
| 334591 | 118 | 118 | 47% | 4e-23 | 99% | BT113356.1 | clone GQ03405_M22 mRNA sequence [*Picea glauca*] |
| 340782 | 41 | 41 | 28% | 3.1 | 100% | XM_010928459.2 | ribokinase [*Elaeis guineensis*] |
| 341940 | 42.8 | 42.8 | 25% | 2.6 | 93% | XM_017585779.1 | probable disease resistance protein At1g12280  [*Vigna angularis*] |
| 505960 | 48.2 | 48.2 | 21% | 0.035 | 84% | BT112618.1 | clone GQ03318_H22 mRNA sequence [*Picea glauca*] |
| 521876 | 158 | 158 | 97% | 3e-35 | 96% | DQ647865.1 | Mitochondrial large subunit ribosomal RNA gene [*Abies homolepis*] |
| 559821 | 42.8 | 42.8 | 48% | 0.88 | 87% | KY110734.1 | DUF21 domain-containing protein At4g14240-like  [Nicotiana attenuata] |
| 638724 | 41 | 41 | 47% | 3.1 | 84% | XM_011032866.1 | cytochrome P450 86B1-like [*Populus euphratica*] |
