# Supplementary figures and images for: Adaptive Genetic Divergence Despite Significant Isolation-by-Distance in Populations of Taiwan Cow-Tail Fir (Keteleeria davidiana var. formosana)
Source: Front Plant Sci. 2018 Feb 1;9:92. doi: 10.3389/fpls.2018.00092 (PMC5799944; doi:10.3389/fpls.2018.00092)

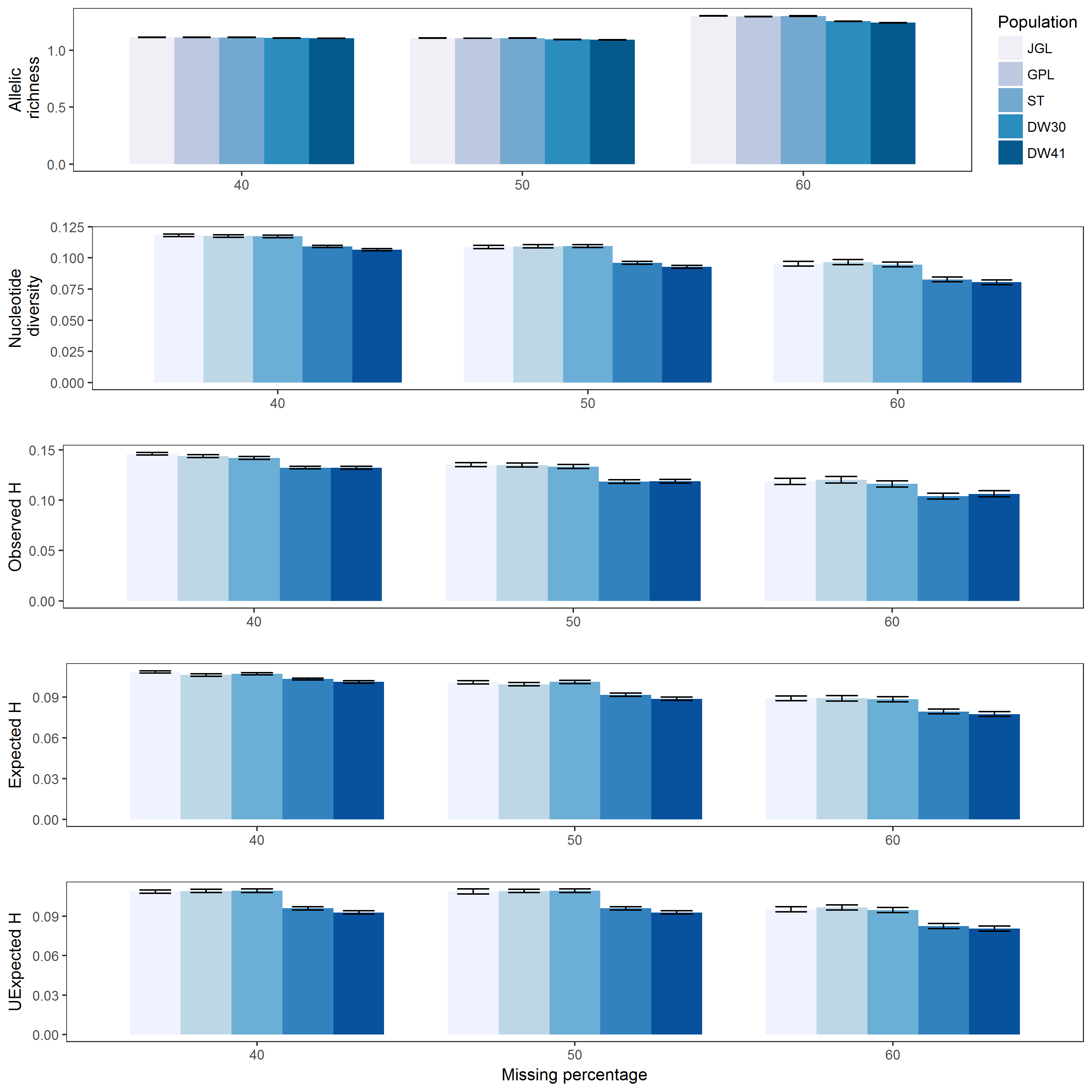

Supplement: Supplementary Figure 1 — Distributions of population genetic diversity measures, including allelic richness, nucleotide diversity, observed heterozygosity, expected heterozygosity, and unbiased expected heterozygosity in data sets of non-missing genotypes in at least 40, 50, and 60% of samples across populations. [file Image1.TIF]

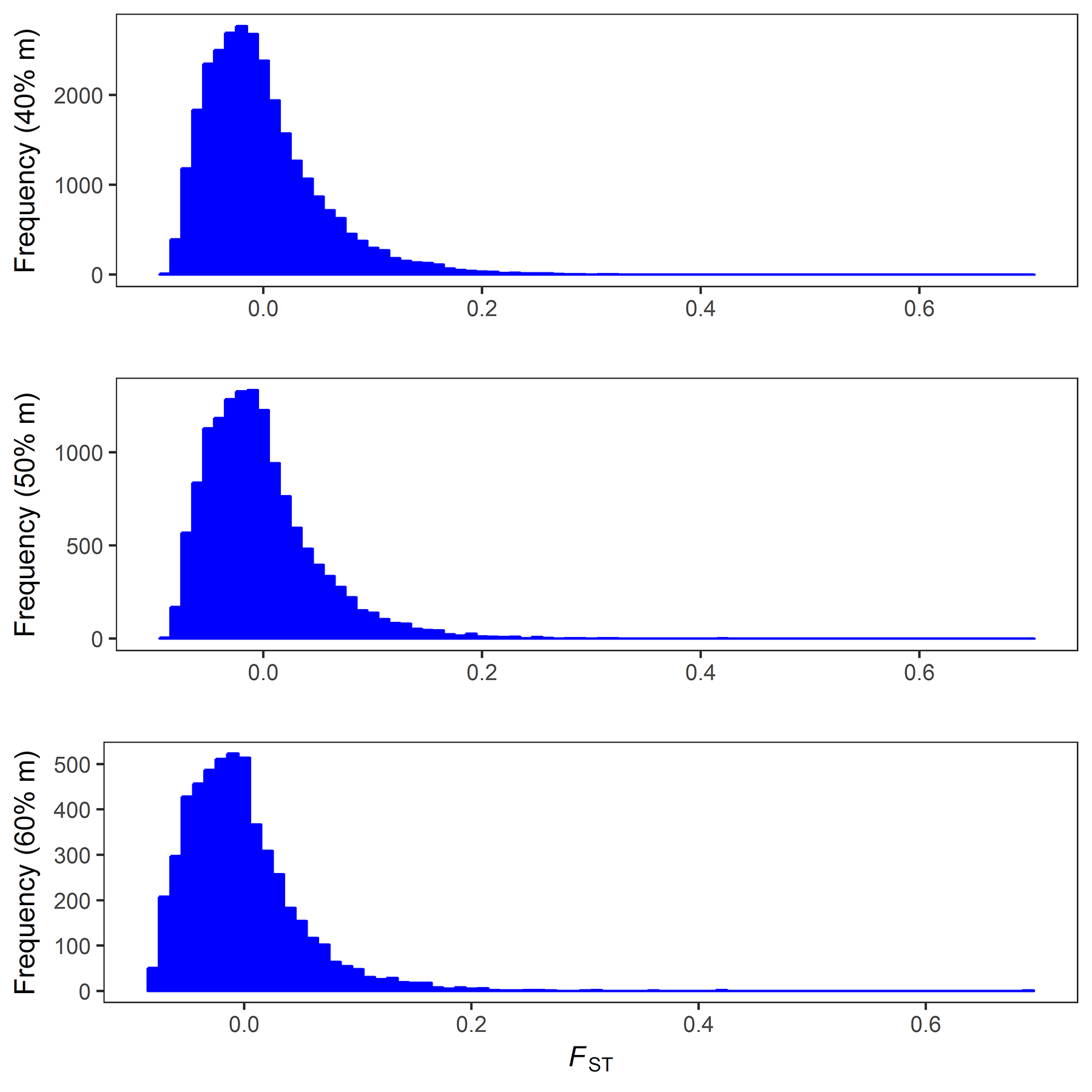

Supplement: Supplementary Figure 2 — Distributions of pairwise locus FST in data sets of non-missing genotypes in at least 40, 50, and 60% of samples across populations. [file Image2.TIF]

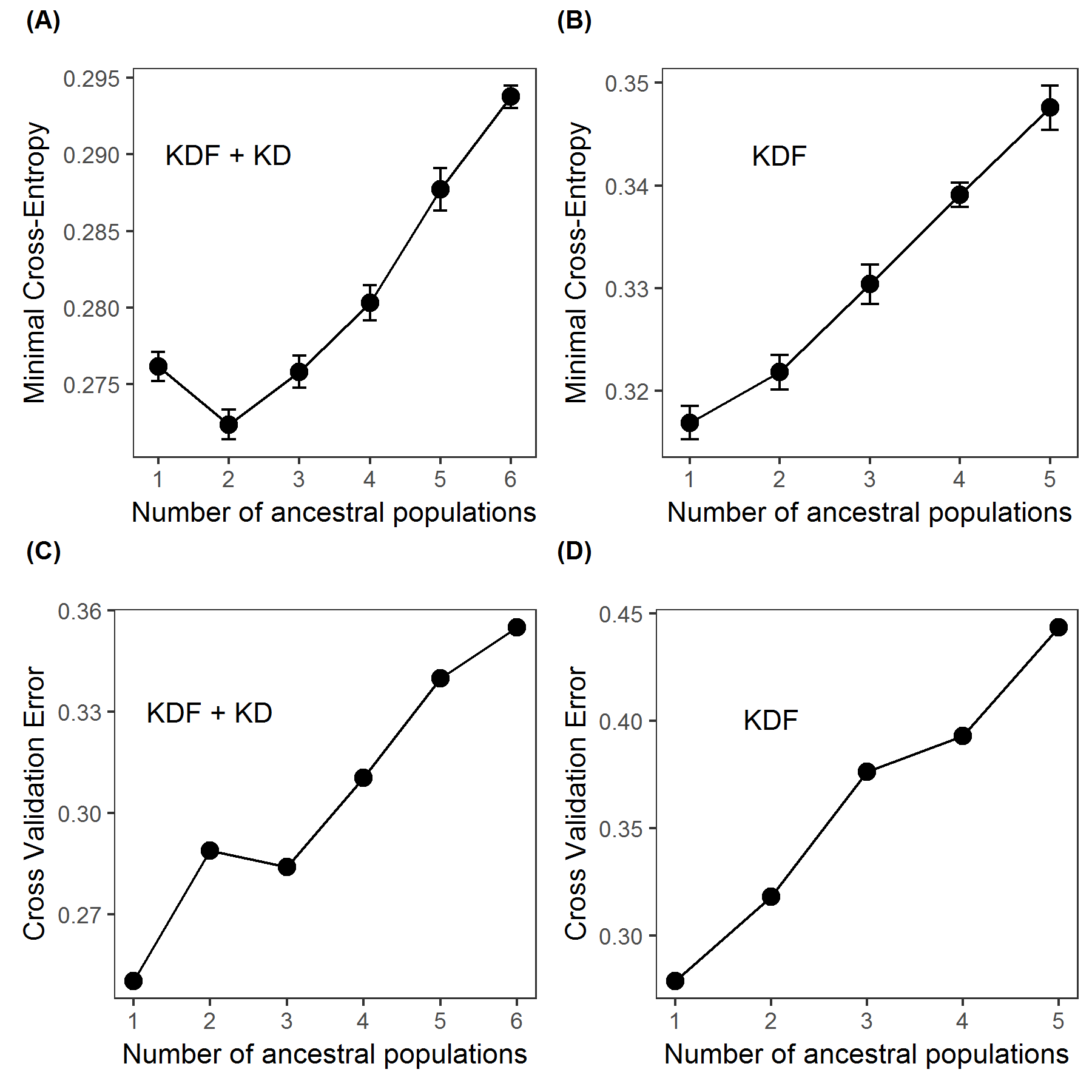

Supplement: Supplementary Figure 3 — Minimal cross-entropy and cross validation error analyzed using LEA and ADMIXTURE. (A,B) LEA and (C,D) ADMIXTURE using samples included both Taiwan cow-tail fir (KDF) and Keteleeria davidiana (KD) or samples of Taiwan cow-tail fir. [file Image3.TIF]
