# Supplementary material for: Adaptive Genetic Divergence Despite Significant Isolation-by-Distance in Populations of Taiwan Cow-Tail Fir (Keteleeria davidiana var. formosana)
Source: Front Plant Sci. 2018 Feb 1;9:92. doi: 10.3389/fpls.2018.00092 (PMC5799944; doi:10.3389/fpls.2018.00092)
Supplement: Supplementary File 1 — A php script for the addition of a 5-bp C and a 5-bp T, respectively, to the end of PE read 1 and read 2. [file Presentation1.PDF]

```php
#!/usr/bin/php
<?php

$dir_path = '/home/d201a/keteleeria/process_reads';

	$selectedfile = list_all_file($dir_path);

	function list_all_file($dir_path)
	{
	$selectedfile = Array();
		if(is_dir($dir_path))
		{
			foreach(scandir($dir_path) as $file)
			{
				if (is_dir($dir_path."/".$file)) continue;

				if($file != '.' && $file != '..')
				{
					//echo $dir_path . '/' . $file."\n";
					//if(substr($file,-3) == ".fq")    $selectedfile[]=$file;
					if(substr($file,-4) == "1.fq")    $selectedfile[]=$file;

				}
			}
		}
		if(is_file($dir_path))
		{
			//echo $dir_path."\n";
		}
		return $selectedfile;
	}


//var_dump($selectedfile);


//exit();
```

```php
$save_path = $dir_path."/fixlength/";


foreach($selectedfile as $s_file){
    var_dump($s_file);
    //$s_file = "A12_1.fq";
    $read_file = $dir_path."/".$s_file;
    $write_file = $dir_path."/fixlength/fix2_".$s_file;

    $handle = fopen($read_file, "r");
    $whandle = fopen($write_file, "w");
    $contents = '';
    if ($handle) {

        $i = 1;
        while (!feof($handle)) {
            /*
            if($i > 500){
                fclose($handle);
                exit();
            }
            */
            $contents = fgets($handle, 255);
            //處理 seq
            if(($i%4) == "2"){
                if(substr($s_file,-4) == "1.fq"){
                    $_seq =
substr("CCCCCCCCCCCCCCCCCCCCCCCCCCCCC".$contents, -83);
                }else{
                    $_seq =
substr("TTTTTTTTTTTTTTTTTTTTTTTTTTTTT".$contents, -83);
                }
                fputs($whandle,$_seq);
            }else if(($i%4) == "0"){
            //處理 qua
                $_qua = substr("iiiiiiiiiiiiiiiiiiiiiiiiiiii".$contents, -83);
                fputs($whandle,$_qua);
            }else{
```

```
                fputs($whandle,$contents);
            }
            //echo $contents;
            $i++;
        }
        fclose($handle);
        fclose($whandle);
    }

}    // end foreach
?>
```
